# The role of body image dissatisfaction in the relationship between body size and disordered eating and self-harm: complimentary Mendelian randomization and mediation analyses

**DOI:** 10.1038/s41380-024-02676-5

**Published:** 2024-08-13

**Authors:** Grace M. Power, Naomi Warne, Helen Bould, Francesco Casanova, Samuel E. Jones, Tom G. Richardson, Jessica Tyrrell, George Davey Smith, Jon Heron

**Affiliations:** 1 MRC Integrative Epidemiology Unit, University of Bristol, Bristol, UK; 2 Population Health Sciences, Bristol Medical School, University of Bristol, Bristol, UK; 3 Institute for Molecular Bioscience, The University of Queensland, Brisbane, QLD Australia; 4 Centre for Academic Mental Health, Population Health Sciences, Bristol Medical School, University of Bristol, Bristol, UK; 5 Gloucestershire Health and Care NHS Foundation Trust, Gloucester, UK; 6 Genetics of Complex Traits, College of Medicine and Health, University of Exeter, Exeter, UK; 7 Institute for Molecular Medicine, University of Helsinki, Helsinki, Finland; 8 NIHR Bristol Biomedical Research Centre Bristol, University Hospitals Bristol and Weston NHS Foundation Trust, University of Bristol, Bristol, UK

**Keywords:** Psychiatric disorders, Genetics, Psychology

## Abstract

Disordered eating and self-harm commonly co-occur in young people suggesting potential for shared underlying causes. Body image dissatisfaction (BID) has been recognised as a psychological correlate of body size, associated with both disordered eating and self-harm. However, the investigation into etiological pathways early in the lifecourse to provide detail on how body size and BID may foster disordered eating and self-harm remains largely unexplored. Employing data from two large population-based cohorts, the UK Biobank and the Avon Longitudinal Study of Parents And Children (ALSPAC), we conducted bidirectional Mendelian randomization (MR) to determine the causal direction of effect between genetically predicted prepubertal body size and two measures of BID indicating (i) desire to be smaller, and (ii) desire to be larger. We then used multivariable regression followed by counterfactual mediation analyses. Bidirectional MR indicated robust evidence that increased genetically predicted prepubertal body size increased desire to be smaller and decreased desire to be larger. Evidence for the reverse causal direction was negligible. These findings remained very similar across sensitivity analyses. In females and males, multivariable regression analyses demonstrated that being overweight increased the risk of disordered eating (risk ratio (RR), 95% confidence interval (CI): 1.19, 1.01 to 1.40 and 1.98, 1.28 to 3.05, respectively) and self-harm (RR, 95% CI: 1.35, 1.04 to 1.77 and 1.55, 0.86 to 2.81, respectively), while being underweight was protective against disordered eating (RR, 95% CI: 0.57, 0.40 to 0.81 and 0.81, 0.38 to 1.73, respectively). There was weak evidence of an increase in the risk of self-harm among underweight individuals. Mediation analyses indicated that the relationship between being overweight and subsequent disordered eating was largely mediated by the desire to be smaller. Our research carries important public health implications, suggesting distinct risk profiles for self-harm and disordered eating in relation to weight and body image. In addition, a better understanding of genetically predicted prepubertal BID may be valuable in the prevention and treatment of disordered eating and self-harm in adolescence.

## Introduction

Incidence rates of eating pathology and self-harm increase during adolescence and are associated with a higher risk of impaired physical health, mortality, and psychiatric comorbidity [1–3]. Whilst phenotypically distinct, disordered eating (weight-control behaviours, abnormal eating) and self-harm (intentionally harming oneself, with or without suicidal intent) have been shown to commonly co-occur in young people, suggesting the likelihood of shared underlying causes [2, 4–6]. Co-occurring disordered eating and self-harm may lead to more severe outcomes than either experience in isolation [4, 7, 8]. Furthermore, those who self-harm often report a history of eating disorders, suicide ideation, and body image dissatisfaction (BID) [9]. BID is defined as a negative subjective evaluation of the weight and shape of one’s own body [10, 11]. Individuals with higher levels of BID have been shown to be at greater risk of eating disorders [12, 13] and self-harm [14].

BID is a potential intervention target in psychological therapy. At the individual-level, BID may be managed effectively to prevent the development of adverse outcomes across an individual’s lifetime. This may be achieved using cognitive-behavioural therapy [15, 16] or self-esteem enhancement [17]. In addition, BID also holds promise as a potentially modifiable risk factor for population-based interventions. These may include media literacy [18, 19] and psychoeducation [20]. The examination of etiologic pathways early in the lifecourse, to provide more detail on how this risk factor fosters both disordered eating and self-harm in adolescence, remains largely unexplored, despite implications for prevention.

Sociocultural explanations of the development of BID propose that society promotes an appearance culture that emphasises the importance of physical attractiveness [21]. Along with emotional well-being and self-esteem, reporting satisfaction with body image is shown to decrease with obesity [22]. The Dual-Pathway model of disordered eating hypothesizes that a higher body mass index (BMI) leads to BID [23]. In addition, studies have shown that the association between BID and body weight is different between sexes [24–26]. Firstly, there appears to be stronger evidence of BID in overweight women in comparison to their normal weight counterparts, than in men [16, 27]. Secondly, boys are more likely to experience BID (utilised as a categorical variable) when they are below or above average weight and most satisfied when they consider themselves an average weight [25]. Conversely, in girls, BID linearly increases with body weight [26]. However, whilst high body weight is seen as a consistent biological factor correlated with BID, whether actual body size causes BID, BID leads to a change in body size, or whether both are occurring at the same time in the peri-pubertal phase, has yet to be determined.

Mendelian randomization (MR) uses genetic variants robustly associated with an exposure of interest to strengthen inference regarding the causal influence of an exposure on an outcome [28, 29]. In theory, this technique is less susceptible to the issues of confounding, including confounding by undiagnosed existing disease (reverse causation), which can affect more conventional epidemiological techniques. A study applying MR in a one-sample setting reported that a standard deviation increase in BMI genetic risk score was associated with a 9% increased odds of BMI (odds ratio (OR): 1.09, 95% confidence interval (CI): 1.01, 1.18) [30]. However, bidirectional MR was not performed and therefore evidence for a potential causal effect of BID on BMI was not determined. In the relationship between body size and disordered eating and self-harm, the role of BID could be as a confounder, where BID could independently lead to both changes in body size and disordered eating or self-harm. BID could also be a mediator, such that high or low body size may cause BID which then leads to increased risk of disordered eating or self-harm. In any single-mediator model there are three posited causal relationships and the direction of all three requires justification. In our case, we focus on the direction of causality between BMI and BID. We believe that reverse causation is unlikely for either path involving disordered eating or-self harm in adolescence since these dependent variables are such that we assume the population does not have the outcome when the mediating variable is measured (disordered eating and self-harm are extremely rare as manifestations of the same phenotypes, prepuberty).

Our work was guided by the hypothesis that prepubertal body size has a causal effect on BID which acts as a mediator between body size and both disordered eating and self-harm. We additionally expected to see an analogous relationship between body size and both disordered eating and self-harm. Using data from two large longitudinal cohorts, the UK Biobank and the Avon Longitudinal Study of Parents And Children (ALSPAC), this investigation set out to assess whether BID may have a causal role in the association between body size and both disordered eating and self-harm in adolescence, by employing a novel combination of epidemiological methods. Specifically, in addition to conventional epidemiological techniques, we used bidirectional two-sample MR to determine the direction of effect between genetically predicted body size and genetically predicted BID. Subsequently, we ran counterfactual mediation analyses accounting for intermediate confounding to infer the causal relationship between body size and both disordered eating and self-harm in adolescence, through the potential mediating effects of prepubertal BID.

## Materials and methods

### Data sources and instruments

#### UK Biobank

We used summary statistics derived from UK Biobank data for MR analyses [31]. Data were collected between 2006 and 2010 on individuals aged between 40 and 69 years old at baseline. Using a prospective cohort study design, data were collected from clinical examinations, assays of biological samples, detailed information on self-reported health characteristics, and genome-wide genotyping [32]. The UK Biobank study has obtained ethics approval from the Research Ethics Committee (REC; approval number: 11/NW/0382) and informed consent from all participants enrolled in UK Biobank.

#### The Avon Longitudinal Study of Parents And Children (ALSPAC)

We obtained data on children and adolescents from ALSPAC, a prospective population-based cohort study that recruited pregnant women living in the former county of Avon (UK) with expected delivery dates between 1 April 1991 and 31 December 1992 [33, 34]. There were 20,248 pregnancies identified as being eligible. The initial number of pregnancies enrolled was 14,541. Of the initial pregnancies, there was a total of 14,676 fetuses, resulting in 14,062 live births and 13,988 children who were alive at 1 year of age. When the oldest children were ~7 years of age, an attempt was made to bolster the initial sample with eligible cases who had failed to join the study originally. The total sample size for analyses using any data collected after the age of seven is therefore 15,447 pregnancies, resulting in 15,658 fetuses. Of these 14,901 children were alive at 1 year of age. The study website contains details of all the data that is available through a fully searchable data dictionary and variable search tool (www.bristol.ac.uk/alspac/researchers/our-data/). Consent for biological samples has been collected in accordance with the Human Tissue Act (2004). Ethical approval for the study was obtained from the ALSPAC Ethics and Law Committee and the local Research Ethics Committee. Informed consent for the use of data collected via questionnaires and clinics was obtained from participants following the recommendations of the ALSPAC Ethics and Law Committee at the time.

#### Weight-based body size—UK Biobank

Genetic variants strongly associated with childhood body size (using *P* ≤ 5 × 10^–8^ for non-false positives of a Type 1 error and *r*^2^ < 0.001 for independence of variants) were identified in a large-scale Genome-Wide Association Study (GWAS), previously undertaken in the UK Biobank study on 453,169 individuals, adjusting for age, sex, and genotyping chip [31]. The childhood body size measure employed recall questionnaire data, involving retrospective responses from adult participants who were asked whether, compared to the average, they were ‘thinner’, ‘about average’, or ‘plumper’, when they were aged 10 years old. This score has been independently validated in three distinct cohorts, providing verification that the genetic instruments can reliably separate childhood body size from adult body size [31, 35, 36].

#### Body size (based on BMI measurements)—ALSPAC

BMI was calculated using height (m) and weight (kg) measured at ALSPAC clinic sessions when participants were aged ~7 (mean age: 7.6 years). Further detail is provided in Supplementary Table 1. We grouped BMI into three body size age-specific categories (underweight (equivalent to the adult BMI cut point, ≤18.5 kg/m^2^), normal weight, and overweight (equivalent to the adult BMI cut point, ≥25 kg/m^2^) [37, 38]).

#### Body image dissatisfaction (BID)—ALSPAC

Perceived and desired body image were evaluated using the Stunkard figure rating scales [39] in a questionnaire administered to children (mean age: 10.7 years). The Stunkard figures are illustrations of different body types ranging from very thin (a value of 1) to very obese (a value of 5; Supplementary Fig. 1) [39]. Children were asked to self-select their perceived and desired body shape from the illustrated figures. BID scores were created by taking the difference between the perceived and desired body images (i.e. a score of zero indicated body satisfaction, whilst a negative score indicated body dissatisfaction (desire to be larger) and a positive score indicated body dissatisfaction (desire to be smaller)).

#### Outcomes—ALSPAC

Self-harm and disordered eating (*fasting* (not eating for at least one day), *purging* (vomiting or taking laxatives/other medications), and *excessive exercise* (that frequently interfered with daily routine/work) for the purpose of losing weight or avoiding gaining weight, as well as *binge-eating* (eating a very large amount of food, with loss of control, in a short period of time)) over the last year were assessed via self-report questionnaire during adolescence (mean age: 16.7 years). A thorough description of these variables as well as the questions that were employed to derive these variables are presented in Supplementary Table 2.

#### Confounders

**Fig. 1:**
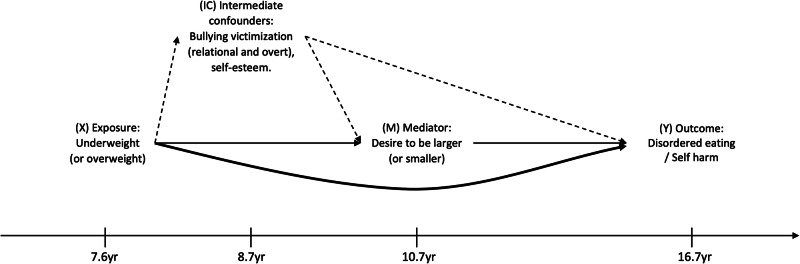
Directed acyclic graph representing associations between prepubertal body size, body image dissatisfaction, and disordered eating and self-harm in adolescence. This graph illustrates the hypothesized role of prepubertal body size and body image dissatisfaction on disordered eating and self-harm in adolescence, and includes the intermediate confounders considered in the main analysis. The thick line indicates a direct effect. The thin lines indicate the indirect pathway. The dotted lines indicate pathways from intermediate confounders. Baseline confounders assumed to confound all paths: Sex; Birthweight; IQ; Indicators of SEP: Maternal education, ethnicity, father absence, weekly income, parent social class, Maternal age; Maternal BMI; Maternal depressive symptoms (32 weeks gestation & 21 months); Stressful life events.

Baseline and intermediate confounders were derived from the literature with support from specialists and clinicians. To guide the modelling strategy, variables were integrated into a directed acyclic graph (DAG) (Fig. 1). Baseline confounders included sex (in analyses that were not sex-stratified), birthweight, IQ, maternal education, ethnicity, father absence, weekly income, parent social class, maternal age, maternal BMI, Edinburgh Postnatal Depression Scale score (32 weeks gestation & 21 months), and stressful life events during childhood assessed using a questionnaire completed by mothers recording whether the child had experienced any of 16 upsetting events since the child was 5 years old. Except for IQ, all baseline confounders were collected at or prior to the data collection period with a mean age of 7.6 years. IQ was measured in a clinic with a mean age of 8.7 years. In including this measure as a baseline confounder, we have made the assumption that IQ did not fluctuate considerably between the ages of ~7 and 9 years. This assumption may not hold if IQ in this age range is affected by body size. Intermediate confounders (confounders of the mediator and outcome association which may be affected by the exposure) comprised self-reported measures of bullying victimization (relational and overt) and self-esteem measured at mean age of 8.7 years. Supplementary Table 3 contains further information on how several of the variables listed were derived and/or defined.

### Statistical analysis

Mediation is reliant on a theoretical model which makes a series of causal assumptions about the links between multiple variables. These causal claims are more challenging when more than one aspect of the model changes in tandem and the direction of causation may be different for different periods of the lifecourse. Thus, we sought to investigate evidence for causality using bidirectional MR to assess the direction of effect between body size and BID (Step 1). Using the appropriate regression model, we determined the confounder-adjusted relationships between exposure, mediator, and outcome (Step 2). Finally, we conducted mediation analyses using *g-formula* to identify the extent to which any causal relationship between body size and both disordered eating and self-harm in adolescence is mediated by prepubertal BID (Step 3). The three key steps are presented in Supplementary Fig. 2 and described in further detail below:

#### Step 1. Genetic analyses

##### Genome-wide Association Studies (GWAS)

We conducted GWAS analyses on 4011 children in ALSPAC who had complete genetic data available for the BID measure collapsed into two binary variables, indicative of (i) dissatisfaction (desire to be larger; *n* = 330) compared to satisfaction and dissatisfaction (desire to be smaller; *n* = 3681) and (ii) dissatisfaction (desire to be smaller; *n* = 938) and satisfaction and dissatisfaction (desire to be larger; *n* = 3073). These were performed using SNPTEST v2.5.6 [40]. Analyses were adjusted for age, sex, and the top 10 genetic principal components and restricted to participants of European ancestry. Children in ALSPAC were genotyped using the Illumina HumanHap550 quad genome-wide SNP genotyping platform. Imputation was performed using Impute V2.2.2 against the 1000 genomes phase 1 version 3 reference panel [41, 42]. Quality control measures included the removal of single nucleotide polymorphisms (SNPs) with ≥5% missing information, deviation from Hardy–Weinberg equilibrium (HWE) (*P* < 1.0 × 10^−6^) and minor allele frequency (MAF) ≤ 1.5% and >99.0% (due to the low case ratio, we increased the allele frequency threshold to exclude SNPs with a low minor allele count in the cases). We generated Manhattan plots which contain horizontal lines drawn at –log10(1 × 10^−5^) for “suggestive associations” and –log10(5 × 10^−8^) for the 'genome-wide significant' threshold and QQ plots (Supplementary Figs. 3 and 4) [43]. We used linkage disequilibrium (LD) clumping with an *r*^2^ threshold of 0.001 to select a set of independent instruments for each trait. This was performed using PLINK [44] and genotype data from European individuals from phase 3 (version 5) enrolled in the 1000 genomes project as a reference panel [45]. There were no genetic variants that reached genome-wide significance (*P* ≤ 5 × 10^–8^) due to the small sample sizes employed (Supplementary Tables 4B and 4C). We relaxed the conventional genome-wide significance *p*-value threshold for instrument selection to *P* ≤ 1 × 10^–4^ for the MR analysis. Applying MR in this study using a Robust Adjusted Profile Score (MR-RAPS) made it possible to employ genetic variants with a less stringent threshold [46, 47].

##### Mendelian randomization (MR)

We ran univariable bidirectional MR analyses to infer the causal direction of body size and BID, by estimating the genetically predicted effect of childhood body size on dissatisfaction (desire to be larger) and dissatisfaction (desire to be smaller) and then dissatisfaction (desire to be larger) and dissatisfaction (desire to be smaller) on childhood body size. We used two non-overlapping cohorts to minimise overfitting.

We used 1689 variants for the childhood body size exposure (Supplementary Table 4A) and 139 and 130 variants for the prepubertal BID (desire to be smaller) and BID (desire to be larger) exposures, respectively (Supplementary Table 4B and 4C).

We used MR-RAPS which maximises the profile likelihood of the ratio estimates and accounts for weak instrument bias, pleiotropy, and extreme outliers [48]. MR-RAPS is shown to be highly efficient with many weak genetic instruments by using an empirical, partially Bayes statistical analysis approach where instruments are weighted according to their strength [47, 48]. Weak instruments therefore bring less variation to the estimator. The inverse variance weighted (IVW) method was then employed as a sensitivity analysis, which takes SNP-outcome estimates and regresses them on the SNP-exposure associations [49]. MR-Egger and Weighted median were subsequently used as additional sensitivity analyses to assess the robustness of the results obtained. MR Egger is more robust to MR assumptions compared to the IVW method, including horizontal pleiotropy, whereby genetic variants influence multiple traits or disease outcomes via independent biological pathways [50].

The MR analyses were conducted using the TwoSampleMR R package [51]. Plots for this study were generated using the R package ‘qqman’ [52]. These analyses were undertaken using R (version 3.5.1).

#### Step 2. Regression analyses—ALSPAC

We estimated the crude and confounder-adjusted associations between body size at age 7 (mean age: 7.6 years) and both disordered eating and self-harm at 16 (mean age: 16.7 years) using Poisson regression to estimate risk ratios. Robust standard errors were used (modified Poisson) as Poisson models for binary outcomes suffer from under-dispersion. Adjusted models were controlled for the baseline confounders described. We then estimated the crude and adjusted relationships between (i) BMI and BID and (ii) BID and both disordered eating and self-harm using the appropriate regression models. We stratified all models by sex. The Likelihood Ratio test was used to measure evidence for effect modification by sex in the effect of BMI on BID.

To mitigate bias due to missing data, we used multiple imputation. This used a Fully Conditional Specification approach [53] with -mi impute chained- in Stata version 16 (StataCorp, 2019), comprising 25 cycles of regression-switching and produced 100 imputed datasets with imputation samples stratified by sex (further detail in Supplementary Table 5).

#### Step 3. Mediation analyses

We estimated the effect of body size on disordered eating and self-harm via BID by conducting parametric g-computation using the Stata package *g-formula* *[*54]. This package uses Monte Carlo simulation to simulate the outcome, mediators, and intermediate confounders under hypothetical interventions or “counter to the fact” scenarios [55].

The total causal effect (TCE) generated using this approach is the value of the outcome (disordered eating or self-harm) if all individuals had been exposed to a one-unit increase in BMI. The natural direct effect (NDE) is the direct (unmediated) effect of BMI on disordered eating or self-harm when BID takes the value it would be in absence of BMI. The natural indirect effect (NIE) is the effect of BMI on disordered eating or self-harm that operates by changing BID. We adjusted all direct and indirect paths for baseline confounders and the intermediate confounding effect. In order for these effects to have a causal interpretation, the following assumptions are required: that there is (i) no unmeasured confounding of the exposure-outcome relationship, (ii) no unmeasured confounding of the mediator-outcome relationship, and (iii) no unmeasured confounding of the exposure-mediator relationship [56].

Preliminary analysis uncovered two sparsely populated cells in the association between the three-category variables representing both BMI and BID—the number of children either being large and wanting to be larger, or small and wanting to be smaller, were in single digits (Supplementary Table 6). Consequently, to mitigate concerns about perfect prediction and prevent computational problems, we decided to split the sample by exposure and conduct two separate mediation analyses. Firstly, we focused on the sample of children who were either classified as normal or underweight and considered the binary BID variable 'desire to be larger' as the mediator. Following this, we switched our attention to the sample who were either considered normal or overweight and treated 'desire to be smaller' as the mediator. In both instances, separate mediation models were estimated for the disordered eating and self-harm outcomes, and for females and males (i.e. eight mediation models in total). Since we only dropped cases based on our exposure variable (BMI), this strategy would not be expected to introduce collider bias. We conducted sex-stratified analyses to provide insights into young male, as well as female, experiences of BID, disordered eating, and self-harm as (i) the published literature suggests experiences differ by sex, and (ii) information of young males in this area is sparse. The same approach to missing data, as described in Step 2, was employed for this section.

All analyses conducted in Steps 2 and 3 used Stata version 16 [57].

## Results

The study sample comprised 2734 (52.6%) females and 2459 males (47.4%). Seven-hundred and thirty-one (22.6%) experienced disordered eating and 347 (10.5%) self-harmed in adolescence. Of those experiencing BID (32.1%), 1219 (73.1%) had a desire to be smaller, and 448 (26.9%) had a desire to be larger. The median BMI for children at age 7 was 15.8 kg/m^2^ (14.9–17.0 kg/m^2^) (Table 1).

**Table 1:** Baseline distribution of selected cohort characteristics (*n* = 5193).

Variable	Category	Total, No.	No. (col%) of participants / median (IQR)
Sex	Female	5193	2734 (52.7%)
	Male		2459 (47.4%)
Birthweight (g)	Median (IQR)	5125	3450 (3130–3760)
Body mass index (kg/m^2^)	Median (IQR)	4829	15.79 (14.85–16.99)
Body image	Satisfaction	5193	3526 (67.9%)
	Dissatisfaction (desire to be larger)		448 (8.6%)
	Dissatisfaction (desire to be smaller)		1219 (23.5%)
Stressful life events	0 events	4632	1631 (35.2%)
	1 event		1485 (32.1%)
	2 events		858 (18.5%)
	3 events		368 (7.9%)
	4 events		184 (4.0%)
	5 events		64 (1.4%)
	6 events or more		42 (0.9%)
IQ	Median (IQR)	5193	105 (94–118)
Disordered eating at 16 years old	Yes	3230	731 (22.6%)
	No		2499 (77.4%)
Self-harm at 16 years old	Yes	3295	347 (10.5%)
	No		2948 (89.5%)
**Characteristics of parents**			
Maternal age (years)	Median (IQR)	5193	29 (26–32)
Maternal body mass index (kg/m^2^)	Median (IQR)	4778	22.14 (20.5–24.2)
Maternal education	No high school qualifications	5087	993 (16.5%)
	High school		1754 (34.5%)
	Beyond high school		2340 (46.0%)
Maternal social class	4–5 (high)	4900	176 (3.6%)
	3 (manual and non-manual)		1616 (33.0%)
	2		2288 (46.7%)
	1 (low)		820 (16.7%)
Paternal absence	No paternal absence	4628	3713 (80.2%)
	Paternal absence when child was 5 years or older		320 (6.9%)
	Paternal absence when child was less than 5 years old		595 (12.9%)
Equivalized income (quintiles)	1	4753	650 (13.7%)
	2		866 (18.2%)
	3		964 (20.3%)
	4		1100 (23.4%)
	5		1173 (24.7%)
Edinburgh Postnatal Depression Scale score (32 weeks gestation)^a^	Median (IQR)	4962	6 (3–10)
Edinburgh Postnatal Depression Scale score (21 months post-partum)^a^	Median (IQR)	4817	4 (2–8)

^a^Mothers scoring above 12 or 13 are likely to be suffering from depression and should seek medical attention.

Of those with BID (desire to be smaller), individuals were more likely to be overweight than normal or low weight. Conversely, those with BID (desire to be larger), were more likely to be under or normal weight (Supplementary Table 7). Experiencing disordered eating was most common in those who were overweight (Table 2A) as well as those with BID (desire to be smaller) than in those with BID (desire to be larger) (Table 2B). These trends were stronger in females than they were in males.

**Table 2:** Disordered eating and self-harm at 16 (mean age: 16.7 years) in adolescents according to body mass index at age 7 (mean age: 7.6 years) or body image at age 10 (mean age: 10.7 years) (imputed data, *n* = 5193).

		Females			Males		
Variable	Category	Total, No. (not imputed)	No. (row %) of adolescents with outcome	Imputed prevalence (95% CI) (imputed data, *n* = 2734)	Total, No. (not imputed)	No. (row %) of adolescents with outcome	Imputed prevalence (95% CI) (imputed data, *n* = 2459)
Models with body mass index at age 7 (mean age: 7.6 years) as the explanatory variable							
**Outcome: Disordered eating**							
Body size	Normal weight	1347	437 (32.4%)	32.9% (30.6–35.5%)	1046	74 (7.1%)	8.3% (6.8–10.3%)
	Underweight	143	26 (18.2%)	18.1% (12.8–25.5%)	99	6 (6.1%)	6.9% (3.3–14.1%)
	Overweight	300	118 (39.3%)	40.6% (35.4–46.4%)	128	23 (18.0%)	17.2% (11.9–24.8%)
**Outcome: Self-harm**							
Body size	Normal weight	1374	185 (13.5%)	13.3% (11.7–15.0%)	1063	57 (5.4%)	5.4% (4.2–6.8%)
	Underweight	149	20 (13.4%)	13.5% (9.1–20.1%)	104	5 (4.8%)	5.5% (2.5–12.0%)
	Overweight	303	51 (16.8%)	19.0% (15.2–23.8%)	136	11 (8.1%)	8.5% (4.9–14.8%)
Models with body image at age 10 (mean age: 10.7 years) as the explanatory variable							
**Outcome: Disordered eating**							
Body image	Satisfaction	1249	367 (29.4%)	29.6% (27.2–32.3%)	971	62 (6.4%)	7.3% (5.8–9.3%)
	Dissatisfaction (desire to be larger)	119	27 (22.7%)	22.2% (16.1–30.8%)	138	12 (8.7%)	9.3% (5.5–15.7%)
	Dissatisfaction (desire to be smaller)	526	227 (43.2%)	43.3% (39.4–47.7%)	227	36 (15.9%)	16.3% (12.2–21.8%)
**Outcome: Self-harm**							
Body image	Satisfaction	1282	162 (12.6%)	13.0% (11.3–15.0%)	989	51 (5.2%)	5.1% (3.9–6.7%)
	Dissatisfaction (desire to be larger)	119	21 (17.7%)	16.6% (11.3–24.5%)	140	10 (7.1%)	7.6% (4.3–13.1%)
	Dissatisfaction (desire to be smaller)	529	87 (16.5%)	16.5% (13.7–19.9%)	236	16 (6.8%)	6.9% (4.4–10.9%)

### Step 1. Genetic analyses (Mendelian randomization)

**Fig. 2:**
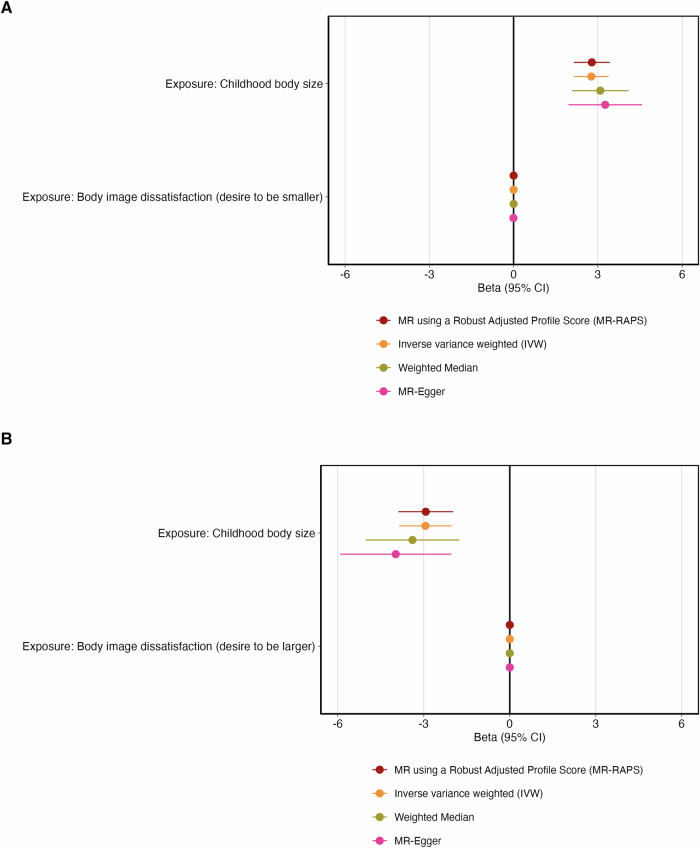
Forest plots illustrating the bidirectional relationships between childhood body size and body image dissatisfaction. **A** Forest plot displaying (i) the effects of childhood body size at age 10 (mean age: 10 years) on body image dissatisfaction (desire to be smaller) at age 10 (mean age: 10.7 years) and (ii) body image dissatisfaction (desire to be smaller) on childhood body size. **B** Forest plot displaying (i) the effects of childhood body size at age 10 (mean age: 10 years) on body image dissatisfaction (desire to be larger) at age 10 (mean age: 10.7 years) and (ii) body image dissatisfaction (desire to be larger) on childhood body size.

Of the children enrolled in ALSPAC of European ancestry, 4011 had complete data available on genotype, BID, age, sex, and the top 10 principal components. Results from MR-RAPS indicated strong evidence that having a larger genetically predicted prepubertal body size was associated with an increased log odds of BID (desire to be smaller) (β: 2.79; 95% CI: 2.15, 3.42; *P* = 1.194 × 10^–17^) (Fig. 2A, Supplementary Table 8A) and a decrease in BID (desire to be larger) (β: –2.93; 95% CI: –3.89, –1.98; *P* = 1.73 × 10^–9^) (Fig. 2B, Supplementary Table 8B). There was very little evidence of the effect of genetically predicted BID (desire to be smaller) and BID (desire to be larger) on prepubertal body size. The effect sizes in this direction were also very small. The directions of effect that were observed using MR-RAPS were reflected in the IVW, Weighted median, and MR-Egger estimates, with differing levels of strength (Fig. 2A, B, Supplementary Tables 8A and 8B).

### Step 2. Regression analyses

Adjusted analyses using imputed data indicated strong evidence in females (*P* < 0.001) and males (*P* = 0.007) that differences in BMI were associated with disordered eating. There was weaker evidence in females (*P* = 0.077) and very little evidence in males (*P* = 0.370) that differences in BMI were associated with self-harm. Females and males who experienced being overweight at age 7 (mean age: 7.6 years) had 1.19 (95% CI: 1.01, 1.40) and 1.98 (95% CI: 1.28, 3.05) times the risk of experiencing disordered eating, respectively, compared to their normal weight counterparts. Females and males experiencing being overweight also had 1.35 (95% CI: 1.04, 1.77) and 1.55 (95% CI: 0.86, 2.81) times the risk of experiencing self-harm, respectively. Further adjusted analyses indicated that females and males who experienced being underweight at age 7 had 0.57 (95% CI: 0.40, 0.81) and 0.81 (95% CI: 0.38, 1.73) times the risk of experiencing disordered eating and 1.09 (95% CI: 0.71, 1.65) and 1.14 (95% CI: 0.50, 2.59) times the risk of experiencing self-harm, respectively, compared to those of normal weight (Table 3).

**Table 3:** Crude and adjusted risk ratios of disordered eating and self-harm at 16 (mean age: 16.7 years) according to body mass index at age 7 (mean age: 7.6 years).

		Females (imputed data, *n* = 2734)		Males (imputed data, *n* = 2459)	
Variable	Category	Crude risk ratio (95% CI)	Adjusted risk ratio (95% CI)^a^	Crude risk ratio (95% CI)	Adjusted risk ratio (95% CI)^a^
**Outcome: Disordered eating**					
Body size	Normal weight	1 [Reference]	1 [Reference]	1 [Reference]	1 [Reference]
	Underweight	0.55 (0.39, 0.78)	0.57 (0.40, 0.81)	0.82 (0.39, 1.73)	0.81 (0.38, 1.73)
	Overweight	1.23 (1.06, 1.44)	1.19 (1.01, 1.40)	2.06 (1.36, 3.14)	1.98 (1.28, 3.05)
	*P* value	<0.001	0.001	0.002	0.007
**Outcome: Self-harm**					
Body size	Normal weight	1 [Reference]	1 [Reference]	1 [Reference]	1 [Reference]
	Underweight	1.02 (0.67, 1.55)	1.09 (0.71, 1.65)	1.01 (0.45, 2.27)	1.14 (0.50, 2.59)
	Overweight	1.44 (1.11, 1.85)	1.35 (1.04, 1.77)	1.59 (0.89, 2.84)	1.55 (0.86, 2.81)
	*P* value	0.019	0.077	0.306	0.370

^a^Adjusted for birthweight, IQ, maternal education, ethnicity, father absence, weekly income, parent social class, maternal age, maternal BMI, the Edinburgh Postnatal Depression Scale (32 weeks gestation & 21 months), and stressful life events during childhood assessed using a questionnaire completed by mothers recording whether the child had experienced any of 16 upsetting events since the child was 5 years.

There was strong evidence that being underweight increased BID (desire to be larger) and being overweight increased BID (desire to be smaller) (Supplementary Table 9). This effect was stronger in male participants, however, whilst there was evidence that the association between BMI and BID varied with sex in unadjusted estimates (*P* = 0.030), there was very little evidence of this when adjusting for selected confounders.

### Step 3. Mediation analyses

In females, the effect of being underweight compared to normal weight on disordered eating was predominantly direct (RR: 0.55; 95% CI: 0.36, 0.83). There is a small proportion of this relationship mediated by BID (desire to be larger) (Table 4A). In contrast, the effect of being overweight compared to normal weight on disordered eating was largely indirect (RR: 1.22; 95% CI: 1.06, 1.41). BID (desire to be smaller) mostly mediated this association (Table 4B). There is suggestive evidence that in males, BID partially mediated the relationship between being underweight and experiencing disordered eating and being overweight and experiencing disordered eating. There is, however, lower power to detect evidence of this effect.

**Table 4:** Decomposition of effects of being underweight or overweight compared to normal on disordered eating and self-harm.

	Females	Males
	Risk ratio (95% CI)	Risk ratio (95% CI)
**Mediation effects when exposure compares underweight to normal weight individuals**		
**Outcome: Disordered eating**		
Total causal effect	0.57 (0.40, 0.81)	0.83 (0.40, 1.73)
Natural direct effect	0.55 (0.36, 0.83)	0.52 (0.20, 1.35)
Natural indirect effect (via body image dissatisfaction (desire to be larger))	1.03 (0.83, 1.29)	1.61 (0.86, 3.04)
**Outcome: Self-harm**		
Total causal effect	1.08 (0.67, 1.74)	1.15 (0.48, 2.78)
Natural direct effect	1.13 (0.67, 1.91)	0.67 (0.24, 1.89)
Natural indirect effect (via body image dissatisfaction (desire to be larger))	0.96 (0.79, 1.17)	1.72 (0.88, 3.36)
**Mediation effects when exposure compares overweight to normal weight individuals**		
**Outcome: Disordered eating**		
Total causal effect	1.19 (1.01, 1.40)	2.01 (0.29, 3.14)
Natural direct effect	0.97 (0.77, 1.22)	1.47 (0.71, 3.06)
Natural indirect effect (via body image dissatisfaction (desire to be smaller))	1.22 (1.06, 1.41)	1.37 (0.80, 2.34)
**Outcome: Self-harm**		
Total causal effect	1.35 (1.02, 1.78)	1.52 (0.82, 2.81)
Natural direct effect	1.39 (0.98, 1.96)	1.15 (0.41, 3.21)
Natural indirect effect (via body image dissatisfaction (desire to be smaller))	0.97 (0.80, 1.19)	1.33 (0.61, 2.86)

There is some evidence to suggest that in females, the effect of being overweight compared to normal weight on self-harm is mostly direct. There was very little mediating effect from BID (desire to be smaller). In males, the effect of being underweight compared to normal on self-harm is mostly indirect, with lower power to detect evidence of this effect. BID (desire to be larger) largely mediated this relationship. The remaining mediation effect estimates obtained reveal partial mediation of BID on the relationship between body size and self-harm.

Due to the opposite sign in the direction of effect in the relationship between the exposure and mediator and the mediator and outcome in some instances, the direct and indirect effect appeared larger than the total effect. This meant that we could not accurately calculate the proportion mediated [58].

## Discussion

In this study, we employed MR and counterfactual mediation to examine the role of BID in the relationship between prepubertal body size and both disordered eating and self-harm in adolescence. Bidirectional MR indicated that higher genetically predicted childhood body size increased BID (desire to be smaller) and decreased BID (desire to be larger). In contrast, there was very little evidence of an effect of genetically predicted BID (a desire to be smaller or larger) on childhood body size.

In regression analyses, being overweight increased risk of disordered eating and self-harm in adolescence in both females and males. Conversely, being underweight appeared to be protective against disordered eating. There was more imprecision and thus weaker evidence for effects seen in males than females since disordered eating and self-harm are less common in adolescent males. The relationship between being overweight and disordered eating was largely mediated by BID (desire to be smaller). The relationship between being overweight and self-harm was only partially mediated by BID (desire to be smaller). There was little evidence of an increase in self-harm in underweight individuals. Our results additionally indicated strong evidence that being underweight increased BID (desire to be larger) and being overweight increased BID (desire to be smaller). The former of these appeared much stronger in males, however, there was little evidence that the association between body size and BID varied by sex upon conducting tests for interaction. Since high comorbidity of disordered eating and self-harm in adolescence has been reported previously [2, 4–6] and common risk factors include increased emotional dysregulation and impulsivity [5, 8], we expected to see an analogous relationship between body size and disordered eating and self-harm. However, our study has shown that risk profiles for disordered eating and self-harm differ in relation to weight-based risk measures.

Published findings regarding the association between being underweight and experiencing self-harm, suicidal ideation or suicide are conflicting. A systematic review and meta-analysis containing 38 observational studies indicated evidence that being underweight increased risk of completed suicide (HR: 1.21, 95% CI: 1.07, 1.36, *p* = 0.002) [59]. On the other hand, a population-based cohort study of male conscripts in Sweden showed that both low and high BMI was associated with increased risk of self-harm [60]. Studies investigating the association between BMI and suicidal ideation have reported different findings for males and females. For example, a study in young adults indicated an association between obesity and suicide risk for women, but not for men [61]. We found that being underweight, as well as overweight, compared to normal weight was more likely to increase self-harm in males relative to females. In addition, the effect of being overweight compared to normal weight on self-harm was only mediated to a small extent by BID (desire to be smaller). An alternative pathway not considered in our study may be that obesity increases risk of depression [62], which could be unrelated to BID. This may also result from the reverse cause, where depression could drive lower weight.

Disordered eating attitudes and behaviours are common among children and adolescents [63]. Those that are overweight during this life stage have been identified as a subset at especially high risk [64]. Whilst binge-eating disorder and night-eating syndrome are forms of disordered eating associated with overweight and obesity, they are also associated with weight gain over time [65, 66]. Binge-eating is reported to have the highest prevalence of comorbid obesity [63]. Results from our MR analyses show a one-directional causal relationship between being overweight and BID.

The majority of studies that have looked at the relationship between body size and body image have done so in female groups [67]. The few previous studies that have examined this relationship in women and men have identified a stronger effect of BID in overweight women compared to their normal weight counterparts, than in men [16, 27]. Conversely, we found a stronger relationship between being overweight and experiencing BID (desire to be smaller) in males than in females. However, tests for interaction indicated that there was very little evidence this association varied by sex. If this discrepancy were indeed real, it may be because the data we used were collected at a time (late 1990s to early 2000s) where changes in cultural attitudes toward the male body have been reported, and males may have felt under increasing pressure to conform to a cultural ideal of a lean, well-toned, muscular build [68]. In addition, counter to previous literature that suggests boys are more likely to experience BID when they are below or above average weight [25, 26], we identified a linear increase of BID with body weight in males as well as females.

This study is an important and novel analysis with several strengths. We used complimentary causal inference epidemiological methods to investigate the role of prepubertal body size and BID on disordered eating and self-harm. Specifically, we used the genetic epidemiological technique, MR (after running GWAS), as well as mediation analyses to assess causal pathways using *g-formula*. We also employed large population-based samples for this research. In addition, we conducted sex-stratified analyses. As a result, this study provides insight into young male experiences of BID, self-harm, and disordered eating which are largely unexplored in the published literature. Lastly, we examined disordered eating and self-harm over a key life period. In focusing on the age groups selected for this study, we were able to observe the exposure and mediator at a life stage where experiences of adolescent manifestations of self-harm and disordered eating had not begun. Intervention prospects in psychological therapy at younger ages for BID to mitigate disordered eating and self-harm in adolescence may additionally be more successful than those conducted in older age groups since the sense of self is still developing and undergoing significant change.

There are, however, limitations to this study. A consequence of the aforementioned strength is that this study is not likely to be generalisable to adult populations where behaviours have already been well-established. Exploration of these experiences in older populations is an important research area and should be independently explored. Disordered eating and self-harm in older adults have distinct characteristics and are associated with a high level of morbidity and mortality [69, 70]. Second, we used an imputed dataset under the assumption that data are missing at random, which, if incorrect, could lead to biased results. However, provided the outcome is unrelated to model missingness, it does not matter whether independent variables are missing not at random [71]. Third, in the ALSPAC sample it is not possible to determine if participants self-defined their disordered eating as self-harm in response to questions on self-harm. Fourth, enrolled ALSPAC participants are from the Southwest of England, more likely to be white, and ineligible for free school meals compared to the National Pupil Database (NPD) ‘Key Stage 4’ (KS4) sample [33]. The results obtained from this study may therefore not be generalisable to other communities in the United Kingdom or indeed globally. Moreover, since allele frequencies, risk factors, and diseases differ between ancestry subgroups, confounding may occur [72]. We have therefore conducted analyses in homogeneous populations of European ancestry and only depict effects within this single ancestry group. Fifth, genetic instruments for childhood body size were derived using recall data and may be more liable to bias. Earlier simulations and validation studies, however, have shown these instruments to effectively separate the effect of childhood body size from adulthood body size [31, 35, 36]. Lastly, due to sample size limitations, the GWAS evaluating BID contained SNPs that did not reach genome-wide significance. However, we were able to run MR-RAPS as the principal MR method. There is potential that the effect estimates for BID on body size may be impacted by weak instrument bias. This would bias the estimates towards the null since the context is a two-sample MR setting (with non-overlapping samples) [73].

Future research in this field would benefit from exploring effects in different age groups to provide more detail on how BID evolves over the lifecourse and how this interacts with disordered eating and self-harm at later stages in life. In addition, whilst novel MR methods are available to perform lifecourse research [74], the current limitation lies in the absence of adequately large datasets containing age-specific information with corresponding genetic data. These data would have the potential to greatly enhance our understanding of this research area.

## Conclusions

This novel application of MR in a counterfactual mediation setting shows a causal effect of prepubertal body size on BID. It additionally suggests distinct risk profiles for disordered eating and self-harm in relation to weight and provides more detail on how risk in early life fosters both disordered eating and self-harm in adolescence. This has important public health implications. A better understanding of the mediating role of genetically predicted prepubertal BID in this context may be useful in the prevention and treatment of disordered eating and self-harm in adolescence.

## Supplementary information

Supplementary Material

## Data Availability

All individual-level data analysed in this study can be accessed via an approved application to ALSPAC (http://www.bristol.ac.uk/alspac/researchers/access/). Summary statistics on prepubertal body size are publicly available from the studies as referenced [31].
