# Supplementary material for: The role of body image dissatisfaction in the relationship between body size and disordered eating and self-harm: complimentary Mendelian randomization and mediation analyses
Source: Mol Psychiatry. 2024 Aug 13;30(2):521–31. doi: 10.1038/s41380-024-02676-5 (PMC11746148; doi:10.1038/s41380-024-02676-5)
Supplement: Supplementary file 1 — Supplementary Material [file 41380_2024_2676_MOESM1_ESM.pdf]

Supplementary Material

Title: The role of body image dissatisfaction in the relationship between body size and disordered eating and self-harm: Complimentary Mendelian randomization and mediation analyses

Running title: Mediating effects of body image dissatisfaction

Author list: Grace M. Power[1,2,3*], Naomi Warne[4], Helen Bould[1,2,4,5], Francesco Casanova[6], Samuel E. Jones[7], Tom G. Richardson[1,2], Jess Tyrrell[6], George Davey Smith[1,2,8#], Jon Heron[1,2,4#]

[1] *MRC Integrative Epidemiology Unit, University of Bristol, Bristol, UK.*

[2] *Population Health Sciences, Bristol Medical School, University of Bristol, Bristol, UK.*

[3] *Institute for Molecular Bioscience, The University of Queensland, Brisbane, Queensland, Australia*

[4] *Centre for Academic Mental Health, Population Health Sciences, Bristol Medical School, University of Bristol, Bristol, UK.*

[5] *Gloucestershire Health and Care NHS Foundation Trust, Gloucester, UK.*

[6] *Genetics of Complex Traits, College of Medicine and Health, University of Exeter, Exeter, UK.*

[7] *Institute for Molecular Medicine. University of Helsinki, Helsinki, Finland*

[8] *NIHR Bristol Biomedical Research Centre Bristol, University Hospitals Bristol and Weston NHS Foundation Trust, University of Bristol, Bristol, UK.*

[#] Joint last authors

[*] Corresponding author: Grace M Power - grace.power@bristol.ac.uk - +44 (0)117 331 0098
MRC Integrative Epidemiology Unit, University of Bristol, Oakfield House, Oakfield Grove, Bristol, BS8 2BN, United Kingdom

Supplementary Table 1. Generating the body size (based on BMI measurements) measure used in this study and age-appropriate international cut points for BMI thresholds by sex between 2 and 18 years (using 7-9.5 years)

We used BMI collected in a younger age band (aged approximately 7 (mean age: 7.6 years)) since this measure came before the intermediary confounders used and mediator of interest, BID. Since we had age at clinic attendance available in whole months, we used interpolation to estimate BMI at age-appropriate international cut points for BMI thresholds by sex (Cole, Bellizzi et al. 2000, Cole, Flegal et al. 2007):

Weight category	Scenario	Process
Overweight	Having a body mass index of ≥25 kg/m^2 is the most widely used international adult cut off point for experiencing being overweight (Cole, Bellizzi et al. 2000). Consequently, child cut off points have been established by sex for ages between 2- and 18-years using dataset specific centiles linked to this adult cut off point.	We used interpolation to estimate age-appropriate international cut points for BMI thresholds by sex. First, we used the age-appropriate cut points available for 6-month windows between 7 and 9.5 years and calculated what these should be per one-month, by sex. We then allocated participants an 'overweight' category if they exceeded these age dependent thresholds.
Underweight	Having a body mass index of ≤18.5 kg/m^2 is the most widely used international adult cut off point for experiencing being underweight (Cole, Flegal et al. 2007). Consequently, child cut off points have been established by sex for ages between 2- and 18-years using dataset specific centiles linked to this adult cut off point.	We used interpolation to estimate BMI at age-appropriate international cut points for BMI thresholds by sex.<br><br>First, we used the age-appropriate cut points available for 6-month windows between 7 and 9.5 years and calculated what these should be per one-month, by sex. We then allocated participants an 'underweight category if they exceeded these age dependent thresholds.

Supplementary Table 2. Disordered eating and self-harm descriptions, questions, variable derivation at 16 years in ALSPAC (taken from Warne et al. (Warne, Heron et al. 2021)).

Disordered eating.
Disordered eating behaviours were measured using the Youth Risk Behaviour Surveillance System questions (Kann, Warren et al. 1996), previously derived by Warne and colleagues (Warne, Heron et al. 2021). Behaviours comprised *fasting* (not eating for at least one day), *purging* (vomiting or taking laxatives/other medications), and *excessive exercise* (that frequently interfered with daily routine/work) for the purpose of losing weight or avoiding gaining weight, as well as *binge-eating* (eating a very large amount of food, with loss of control, in a short period of time). Behaviours were considered present if they were reported at any frequency in the last year. The primary variable of interest was any disordered eating (any of the aforementioned behaviours).<br><br>Any Disordered Eating = Any Fasting OR Any Purging OR Any Excessive Exercise OR Any Binge-eating

Questions	Response options	Coding	Final variable
	Fasting		
During the past year, how often did you fast (not eat for at least a day) to lose weight or avoid gaining weight?	1. Never<br>2. Less than once a month<br>3. 1-3 times a month<br>4. Once a week<br>5. More than once a week	Any fasting<br>1 = "no"<br>2-5 = "yes"	Any fasting
	Purging		
During the past year, how often did you make yourself throw up (vomit) to lose weight or avoid gaining weight?	1. Never<br>2. Less than once a month<br>3. 1-3 times a month<br>4. Once a week<br>5. 2-6 times a week<br>6. Every day	Any self-induced vomiting<br>1 = "no"<br>2-6 = "yes"	Any purging<br><br>Any self-induced vomiting<br>OR<br>Any laxative/medication use
a) During the past year, did you take laxatives or other tablets or medicines (diet pills or water tablets) to lose weight or avoid gaining weight?<br><br>b) How often?	1. Yes, laxative<br>2. Yes, other<br>3. Never<br><br>1. Never<br>2. Less than once a month<br>3. 1-3 times a month<br>4. Once a week<br>5. 2-6 times a week<br>6. Every day	Any laxative/medication use<br>a) 3 = "no"<br>a) 1-2 = "yes"<br>OR<br>b) 2-6 = "yes"	

Questions	Response options	Coding	Final variable
Excessive exercise			
During the past year, how often did you do any exercise (going to the gym, brisk walking or any sports activity)?	1. 5 or more times a week<br>2. 1-4 times a week<br>3. 1-3 times a month<br>4. Less than once a month<br>5. Never	Any exercise<br>5 = “no”<br>1-4 = “yes”	Any excessive exercise<br><br>Any exercise to lose weight<br>AND<br>Exercise interfered with life<br>(= 0 if Any exercise = “no”)
Did you exercise in order to lose weight or avoid gaining weight?	1. Yes, sometimes<br>2. Yes, frequently<br>3. No	Any exercise to lose weight<br>3 = “no”<br>1-2 = “yes”	
Was it difficult for you to do your work or schoolwork because of the amount of time that you were exercising?	1. Yes, sometimes<br>2. Yes, frequently<br>3. No	Exercise interfered with life<br>1 & 3 = “no”<br>2 = “yes”	
Binge eating			
Sometimes people will go on an ‘eating binge’, where they eat an amount of food that most people would consider to be very large, in a short period of time. During the past year, how often did you go on an eating binge?	1. Less than once a month<br>2. 1-3 times a month<br>3. Once a week<br>4. More than once a week<br>5. Never	Any bingeing<br>5 = “no”<br>1-4 = “yes”	Any binge-eating<br><br>Any bingeing<br>AND<br>Loss of control
Did you feel out of control, like you couldn’t stop eating even if you wanted to?	1. Yes, usually<br>2. Yes, sometimes<br>3. No	Loss of control<br>3 = “no”<br>1-2 = “yes”	

Self-harm.

For *self-harm*, participants were asked a set of questions adapted from the Child and Adolescent Self-Harm in Europe study (Madge, Hewitt et al. 2008), about whether they had hurt themselves on purpose in any way (regardless of suicidal intent), and when this occurred during the last year. Self-harm was considered present if it was reported as occurring in the past year (regardless of suicidal intent). Questions and variable coding are available elsewhere (Madge, Hewitt et al. 2008).

Self-harm			
When was the last time you hurt yourself on purpose?	1. In the last week<br>2. More than a week ago but in the last year<br>3. More than a year ago	Self-harm past year<br>3 = “no”<br>1-2 = “yes”	Self-harm<br>When was the last time you hurt yourself on purpose?
Have you ever hurt yourself on purpose in any way (e.g. by taking an overdose of pills, or by cutting yourself)?	1. Yes<br>2. No	Self-harm ever<br>1 = “yes”<br>2 = “no”	

Supplementary Table 3. Information on confounders and intermediate confounders

Name of confounder and intermediate confounder	Further detail	Type
IQ	Full scale IQ was assessed at age 8 years in a face-to-face clinic session using the Wechsler Intelligence Scale for Children-III (WISC-III) (Wechsler 1991).	Confounder
Indicators of socioeconomic position	Indicators of socioeconomic disadvantage included parent social class (reported during pregnancy); maternal education (reported at 32 weeks' gestation as mother's highest educational qualification (coded as no high school qualifications, high school, beyond high school); average weekly income (quintiles measured on maternal questionnaires at 33 and 47 months); paternal absence (coded no paternal absence, paternal absence when child was 5 years or older, paternal absence when child was less than 5 years old); and maternal age at delivery.	Confounder
Maternal depressive symptoms	Maternal depressive symptoms were reported on the Edinburgh Postnatal Depression Scale (EPDS) at 32 weeks of gestation and at 21 months post-partum (Cox, Holden et al. 1987). These variables were included in the model as two continuous variables.	Confounder
Stressful life events	Stressful life events which occurred when the child was 7-8 years old were reported by main carer via questionnaire when the study child was 8.5 years (Warne, Heron et al. 2023). Carers were asked whether selected events had occurred and the impact of these following the child's 7th birthday. These included: being taken into care; a pet dying; moving home; being physically hurt by someone; being sexually abused; having a family member die; separation from mother, father, or someone else; having a new mother, father or sibling; being admitted to hospital; having a change in caretaker; starting a new school; and losing a best friend. We treated this as a scale, only collapsing observations at the top end (6 or more was recoded as 6).	Confounder
Bullying victimisation	At mean age 12 years 10 months, participants indicated the presence and frequency of nine (overt and relational) bullying behaviours experienced over the past 6 months on the Bullying and Friendship Interview Schedule (Wolke, Woods et al. 2001). Item responses were	Intermediate confounder

	rated on a scale of "never" (0), "seldom (<4 times)" (1), "frequently (≥4 times)" (2), or "very frequently (at least once a week)" (3). We summed responses to create a continuous score (range 0-27) (Bowes, Joinson et al. 2015). These variables were included in the model as two separate variables.	
Self-esteem	At mean age 8.5 years, participants completed a shortened form of Harter's Self Perception Profile for Children (Harter 1985) as part of the face-to-face assessment clinic (Fisher, Schreier et al. 2012). This variable was included in the model as a continuous variable.	Intermediate confounder

Supplementary Table 4A. Genome-wide association study results for early life body size in the UK Biobank

The sets of genetic variants used may be obtained from the reference listed below. For the sake of conserving space, they have not been listed here.

Trait	Units	Ref.
Childhood body size	Body size category	Richardson T G, Sanderson E, Elsworth B, Tilling K, Davey Smith G. Use of genetic variation to separate the effects of early and later life adiposity on disease risk: mendelian randomisation study. *BMJ*. 2020; 369 :m1203 doi:10.1136/bmj.m1203

Supplementary Table 4B. Genome-wide association study results for dissatisfaction (desire to be smaller) in ALSPAC

SNP	CHR	BP	ALLELE1	ALLELE0	A1FREQ	BETA	SE	P
rs1411402	1	10579545	T	G	0.33	-0.23	0.06	6.54E-05
rs12126578	1	107490554	C	A	0.47	0.23	0.05	1.82E-05
rs34291690	1	120099137	A	G	0.21	0.27	0.07	4.78E-05
rs4950361	1	146559165	A	G	0.30	0.24	0.06	2.15E-05
rs150963900	1	156347147	G	C	0.02	-1.02	0.29	7.69E-05
rs61814526	1	181528371	T	C	0.04	-0.66	0.17	3.93E-05
rs116407970	1	198918084	T	C	0.02	-1.22	0.28	8.20E-07
rs1411404	1	209219452	T	A	0.61	-0.24	0.06	1.01E-05
rs1556891	1	217168377	C	A	0.62	0.24	0.05	1.36E-05
rs2143101	1	22578436	A	G	0.26	-0.25	0.06	8.46E-05
rs146747561	1	32854745	T	C	0.03	-0.80	0.21	3.81E-05
rs12405341	1	4000041	C	G	0.10	0.39	0.09	1.20E-05
rs143914389	1	55105614	C	G	0.05	0.59	0.12	5.70E-07
rs12062838	1	55442357	G	A	0.41	-0.26	0.06	4.19E-06
rs116556071	1	6687748	T	C	0.02	0.69	0.17	6.25E-05
rs141632836	1	88692313	A	G	0.03	0.68	0.15	1.51E-05
rs3100713	2	138702469	T	C	0.84	0.31	0.08	6.88E-05
rs72861326	2	144524077	C	T	0.12	-0.37	0.09	2.03E-05
rs262269	2	180884495	G	A	0.78	0.29	0.07	1.75E-05
rs114838626	2	190477498	C	G	0.03	-0.82	0.22	3.06E-05
rs10176569	2	210175008	G	A	0.51	0.21	0.05	6.88E-05
rs13415274	2	21943572	C	T	0.03	-0.95	0.22	1.08E-06
rs35812841	2	234173270	G	C	0.67	-0.23	0.06	6.03E-05
rs1379038	2	41075154	T	A	0.17	0.30	0.07	3.58E-05
rs7593413	2	52018424	G	T	0.08	0.37	0.09	9.00E-05
rs62163128	2	85958645	C	T	0.08	0.41	0.09	1.21E-05
rs9848728	3	101673386	A	G	0.35	0.22	0.06	7.66E-05
rs9289754	3	147342226	C	T	0.06	-0.58	0.15	2.86E-05
rs12233616	3	186610114	A	G	0.22	-0.30	0.07	1.17E-05
rs67013638	3	19230451	G	T	0.52	-0.23	0.06	2.70E-05
rs7623969	3	22058203	T	C	0.06	-0.50	0.13	6.57E-05
rs1478839	3	35083031	C	T	0.22	-0.26	0.07	8.20E-05
rs13100652	3	41710889	T	C	0.11	0.35	0.08	5.28E-05
rs4686252	3	8327822	A	C	0.56	0.23	0.06	5.20E-05
rs79632829	4	13094889	C	T	0.02	-0.91	0.26	7.16E-05
rs112020395	4	140784710	A	G	0.03	0.60	0.14	4.84E-05
rs115610811	4	163301009	A	G	0.03	0.65	0.16	6.69E-05
rs11737546	4	183063383	C	G	0.31	0.24	0.06	4.27E-05
rs74834068	4	38490374	A	C	0.14	-0.35	0.08	1.75E-05
rs116497261	4	49091044	C	T	0.02	1.00	0.22	7.26E-06
rs145475731	4	8075735	T	A	0.05	-0.57	0.14	1.46E-05

rs74676984	5	108667194	C	T	0.07	-0.57	0.13	2.01E-06
rs1122968	5	117930282	C	A	0.42	-0.24	0.06	1.41E-05
rs34371503	5	138001263	A	G	0.02	0.73	0.17	2.56E-05
rs193552	5	148172599	T	A	0.56	0.23	0.06	4.62E-05
rs113618332	5	165270909	G	A	0.02	-1.12	0.32	6.68E-05
rs115229938	5	166331271	T	C	0.03	-0.86	0.20	3.58E-06
rs185394018	5	34708597	C	G	0.02	0.78	0.19	6.76E-05
rs76507295	5	6619016	A	G	0.03	-0.88	0.21	3.98E-06
rs11958666	5	67092939	A	G	0.09	-0.41	0.10	4.35E-05
rs12055120	5	79647809	G	C	0.17	-0.30	0.08	5.38E-05
rs9322787	6	104520286	T	G	0.37	0.23	0.06	4.81E-05
rs140305792	6	134399635	G	A	0.02	-1.10	0.29	1.43E-05
rs7769903	6	137607358	T	C	0.63	0.23	0.06	7.56E-05
rs16900377	6	158149648	T	C	0.02	0.74	0.17	2.39E-05
rs35073086	6	168074339	T	C	0.14	-0.47	0.12	8.85E-05
rs115471279	6	42422788	C	T	0.02	0.83	0.20	4.89E-05
rs11961412	6	81051896	G	A	0.05	0.46	0.11	5.09E-05
rs12194163	6	8511306	G	A	0.02	-1.20	0.32	1.09E-05
rs2470939	7	104581510	A	G	0.39	0.26	0.05	2.23E-06
rs9886021	7	110419943	T	G	0.15	-0.34	0.08	1.09E-05
rs9639594	7	29179186	A	G	0.20	-0.31	0.07	9.98E-06
rs76386730	7	41204522	A	T	0.05	0.51	0.13	7.29E-05
rs4718347	7	65904888	G	A	0.05	-0.69	0.16	2.92E-06
rs1860532	7	8151884	G	A	0.51	-0.23	0.05	3.22E-05
rs4141151	7	83737361	G	A	0.79	0.28	0.07	4.18E-05
rs117543693	8	102370578	G	T	0.02	0.67	0.17	8.12E-05
rs10108740	8	11151049	G	C	0.05	0.56	0.11	1.98E-06
rs2270409	8	141105774	A	G	0.22	0.27	0.06	1.93E-05
rs7017240	8	20147015	A	C	0.77	-0.27	0.06	1.33E-05
rs2241989	8	401789	C	A	0.15	-0.32	0.08	5.22E-05
rs2599683	8	40450143	T	A	0.30	-0.24	0.06	6.44E-05
rs7013487	8	72179992	T	C	0.04	-0.66	0.18	7.34E-05
rs117061826	8	80230543	A	G	0.07	-0.51	0.13	4.84E-05
rs56328659	8	89502092	C	G	0.11	-0.38	0.09	2.17E-05
rs10760670	9	101872163	A	G	0.21	0.25	0.06	9.22E-05
rs117148884	9	109223112	A	G	0.06	-0.51	0.13	3.32E-05
rs72752137	9	118895751	A	G	0.32	-0.34	0.06	8.12E-08
rs1747839	9	139015411	G	T	0.17	-0.29	0.08	8.45E-05
rs56020478	9	139892180	G	C	0.09	-0.50	0.11	4.38E-06
rs149213949	9	24666389	C	T	0.12	0.33	0.08	3.92E-05
rs76708337	9	29537825	A	C	0.02	0.74	0.18	6.86E-05
rs72729115	9	74952658	A	C	0.03	-0.73	0.20	5.49E-05
rs79156891	10	117944736	C	T	0.02	0.87	0.20	1.61E-05

rs491299	10	131750134	T	C	0.59	0.26	0.06	4.78E-06
rs143518880	10	134865329	A	G	0.04	0.52	0.13	9.64E-05
rs111512885	10	16595073	T	C	0.03	-0.84	0.23	6.86E-05
rs17595815	10	36031676	C	A	0.17	0.28	0.07	9.48E-05
rs7917895	10	54453387	A	G	0.13	-0.34	0.09	3.67E-05
rs876792	12	13430216	C	T	0.67	-0.27	0.06	1.20E-06
rs80086846	12	80604767	G	A	0.02	0.68	0.17	7.43E-05
rs79738065	12	81102758	A	C	0.03	-0.76	0.20	3.78E-05
rs139064369	12	99410995	A	G	0.02	-1.03	0.29	4.99E-05
rs778327	13	106152902	T	C	0.69	0.27	0.06	5.47E-06
rs150413827	13	111915011	T	C	0.02	0.72	0.17	5.09E-05
rs3211770	13	113793849	A	G	0.11	0.33	0.08	6.58E-05
rs4624009	13	21687916	T	A	0.24	0.25	0.06	7.87E-05
rs2573265	13	46510181	T	G	0.61	-0.22	0.05	7.41E-05
rs2027542	13	54074825	G	T	0.14	0.31	0.07	5.62E-05
rs117033565	13	71559675	C	T	0.02	0.70	0.16	1.08E-05
rs72633512	13	86509585	C	A	0.02	-1.40	0.32	2.61E-07
rs66523331	13	95880950	A	G	0.20	0.26	0.06	5.81E-05
rs78113926	14	104337460	C	T	0.06	0.43	0.11	9.03E-05
rs118024818	14	28247127	T	C	0.16	0.29	0.07	7.50E-05
rs1952792	14	38324840	C	G	0.95	0.56	0.14	2.32E-05
rs59135299	14	78038421	A	G	0.05	0.45	0.11	6.74E-05
rs10138154	14	92132138	T	C	0.39	0.23	0.05	3.26E-05
rs146321950	14	96945412	T	A	0.04	0.58	0.14	7.61E-05
rs147369302	14	98067555	A	C	0.02	-1.07	0.31	7.97E-05
rs11161349	15	24020129	C	G	0.29	-0.27	0.06	1.16E-05
rs75391484	15	56453138	C	T	0.02	-1.02	0.27	2.69E-05
rs144964918	15	67002822	G	A	0.02	0.77	0.19	7.10E-05
rs4381552	15	80315416	T	G	0.62	0.23	0.06	4.77E-05
rs1905232	15	81843363	G	A	0.06	-0.53	0.13	1.68E-05
rs28638153	15	94689756	C	A	0.27	-0.24	0.06	8.90E-05
rs8064205	16	369413	A	G	0.50	-0.21	0.05	6.53E-05
rs13380427	16	52456114	A	G	0.08	0.38	0.09	6.72E-05
rs11640166	16	52925622	A	T	0.31	-0.25	0.06	3.54E-05
rs62033400	16	53811788	G	A	0.39	0.23	0.05	2.80E-05
rs3829516	16	84777926	T	C	0.86	0.39	0.08	1.43E-06
rs4073992	17	1845462	T	A	0.31	0.22	0.06	9.82E-05
rs8065748	17	26270608	C	A	0.14	0.30	0.07	5.41E-05
rs79811178	17	43108208	A	G	0.07	0.40	0.10	4.82E-05
rs62082874	17	58832105	C	G	0.41	0.47	0.10	5.65E-07
rs72843127	17	65756235	C	A	0.19	-0.38	0.08	3.21E-06
rs9302983	17	66786581	G	T	0.74	0.25	0.06	4.79E-05
rs139695506	18	30155642	A	C	0.02	0.78	0.18	2.90E-05

rs7506696	18	4710695	C	A	0.16	0.30	0.07	3.73E-05
rs141763796	18	55748548	T	C	0.17	-0.35	0.08	7.51E-06
rs4805123	19	35870780	T	A	0.27	-0.24	0.06	9.78E-05
rs11671874	19	58748851	C	T	0.21	0.26	0.06	5.04E-05
rs6080834	20	17775259	A	G	0.26	0.25	0.06	2.62E-05
rs6068918	20	53237206	T	A	0.03	-0.66	0.18	8.15E-05
rs1741605	20	62142483	T	C	0.12	0.36	0.08	8.07E-06
rs2297279	21	42742464	A	G	0.03	-0.79	0.20	1.27E-05
rs134795	22	27668544	A	G	0.40	-0.25	0.06	4.53E-06
rs73160052	22	37474521	A	G	0.05	-0.58	0.15	3.06E-05
rs2072853	22	43533422	A	G	0.02	-0.93	0.24	1.21E-05
rs9627876	22	49548341	T	C	0.21	0.30	0.07	1.72E-05

SNP: Single nucleotide polymorphism; CHR: Chromosome; BP: Base pair position; A1FREQ: Alle frequency of allele 1; Beta: Effect size of allele 1; SE: Standard error; P: P value

Supplementary Table 4C. Genome-wide association study results for dissatisfaction (desire to be larger) in ALSPAC

SNP	CHR	BP	ALLELE1	ALLELE0	A1FREQ	BETA	SE	P
rs17474549	1	108028617	C	A	0.05	-1.02	0.28	2.02E-05
rs67825981	1	119669318	C	T	0.04	-1.08	0.32	8.61E-05
rs2013089	1	15479577	G	C	0.79	-0.39	0.09	5.28E-05
rs2422349	1	173340809	T	G	0.73	-0.37	0.09	4.27E-05
rs4951158	1	205011659	C	T	0.11	0.48	0.12	9.34E-05
rs10863668	1	222090944	A	G	0.30	0.34	0.09	9.71E-05
rs111434736	1	245226145	T	A	0.04	-1.31	0.40	7.94E-05
rs7533885	1	5278635	C	G	0.49	0.34	0.08	3.93E-05
rs138886086	1	72170643	G	A	0.02	1.11	0.24	3.28E-05
rs76211710	1	77532595	G	A	0.02	0.92	0.21	5.75E-05
rs17398377	1	94590939	C	T	0.27	0.36	0.09	6.07E-05
rs35999235	2	101364631	G	T	0.16	-0.48	0.13	8.91E-05
rs62152943	2	107286397	A	G	0.03	-1.61	0.48	9.69E-06
rs77823120	2	11314063	T	A	0.03	-1.51	0.49	7.98E-05
rs12328135	2	142706544	T	C	0.02	-1.84	0.60	3.88E-05
rs115476031	2	175714474	G	C	0.02	-2.64	1.04	7.18E-05
rs78310677	2	186006943	G	A	0.02	1.12	0.25	6.06E-05
rs149950918	2	189099820	T	C	0.02	-2.63	0.99	4.71E-05
rs10165217	2	233843826	C	T	0.80	-0.45	0.09	2.24E-06
rs7580081	2	25097072	C	G	0.45	-0.36	0.08	1.87E-05
rs75890190	2	30552223	T	G	0.02	1.26	0.26	8.33E-06
rs112882319	2	49425329	T	C	0.05	-1.00	0.28	4.97E-05
rs113334486	2	58542622	A	G	0.16	-0.56	0.14	1.15E-05
rs10173093	2	8642499	A	G	0.02	1.01	0.23	6.41E-05
rs2697160	3	10975251	G	A	0.71	-0.34	0.09	9.05E-05
rs145814095	3	11223539	T	G	0.02	-2.67	1.08	6.63E-05
rs9990359	3	116129916	G	C	0.12	-0.59	0.16	5.70E-05
rs57512309	3	134399344	T	G	0.26	0.37	0.09	3.94E-05
rs3749446	3	183586005	T	C	0.68	0.36	0.09	9.20E-05
rs7620153	3	197259920	C	G	0.18	0.40	0.10	5.41E-05
rs115057071	3	24276797	T	C	0.02	-1.79	0.58	3.52E-05
rs2542387	3	9651610	G	A	0.96	1.22	0.35	2.62E-05
rs73144644	3	99203229	A	G	0.07	0.58	0.14	7.10E-05
rs138895539	4	121798710	G	A	0.02	1.05	0.22	1.95E-05
rs10516615	4	122974607	G	A	0.14	-0.56	0.14	2.54E-05
rs114210379	4	136682024	A	G	0.02	-1.78	0.61	8.01E-05
rs72724722	4	140020438	T	C	0.10	-0.70	0.19	5.11E-05
rs13105536	4	164570672	A	G	0.27	-0.40	0.10	4.53E-05
rs78420982	4	19859954	T	A	0.03	0.86	0.19	2.38E-05
rs13101764	4	20834563	T	G	0.02	-1.81	0.59	3.11E-05
rs12647040	4	32385070	C	T	0.15	-0.54	0.14	2.34E-05

rs73241314	4	36831333	G	A	0.16	0.49	0.11	8.54E-06
rs1839364	4	62027123	A	G	0.02	-1.70	0.58	9.06E-05
rs58167498	4	696644	G	A	0.04	0.87	0.18	6.72E-06
rs78270071	4	72188095	G	A	0.02	-4.04	1.79	1.24E-05
rs139429587	4	729741	A	G	0.02	1.00	0.23	9.58E-05
rs11731447	4	77604925	A	C	0.08	-0.78	0.21	2.74E-05
rs114930077	4	82740611	T	C	0.02	-2.12	0.75	6.27E-05
rs2403326	5	103791044	G	A	0.48	0.33	0.08	7.19E-05
rs6555693	5	165869550	T	G	0.64	-0.35	0.09	3.97E-05
rs7727718	5	173171949	C	A	0.13	-0.63	0.15	7.80E-06
rs113273992	5	31654170	T	C	0.03	-1.57	0.49	4.56E-05
rs76874290	6	106838377	C	T	0.13	0.45	0.11	7.13E-05
rs9392258	6	1149856	C	T	0.16	0.41	0.10	9.64E-05
rs13212719	6	133835436	G	A	0.18	0.43	0.10	1.63E-05
rs13204138	6	145033615	C	T	0.07	0.60	0.14	4.14E-05
rs9386240	6	149279188	C	T	0.21	-0.43	0.11	7.29E-05
rs657702	6	150746883	C	G	0.03	-1.46	0.47	7.52E-05
rs9294952	6	169485044	G	C	0.47	0.34	0.08	5.27E-05
rs1033872	6	19673765	A	G	0.26	-0.41	0.10	3.88E-05
rs9267657	6	31841618	G	A	0.03	-1.43	0.46	7.80E-05
rs76402700	6	68460537	A	G	0.02	1.18	0.24	8.99E-06
rs9502693	6	8216901	G	A	0.24	0.36	0.09	7.27E-05
rs2237777	7	126642455	A	C	0.35	-0.42	0.09	2.72E-06
rs62490874	7	129170455	A	G	0.10	-0.62	0.17	5.95E-05
rs10950521	7	14429350	T	C	0.71	0.36	0.10	9.38E-05
rs62448763	7	48712206	A	G	0.05	0.64	0.15	5.66E-05
rs78167286	7	86752268	T	G	0.08	-0.83	0.22	3.84E-05
rs3750232	8	125570528	G	A	0.57	0.35	0.09	3.55E-05
rs72735718	8	135471124	A	G	0.02	-1.87	0.64	6.54E-05
rs11777642	8	20328099	G	C	0.03	-1.90	0.60	1.83E-05
rs13269886	8	27628308	A	T	0.04	0.79	0.19	8.61E-05
rs62481639	8	3804514	A	G	0.30	0.35	0.09	5.74E-05
rs2594781	8	55184094	C	G	0.40	0.33	0.08	6.87E-05
rs10095331	8	6913850	G	A	0.79	-0.38	0.10	9.35E-05
rs2044742	8	76595289	A	G	0.13	-0.55	0.15	7.25E-05
rs76881789	9	113373231	A	G	0.02	-1.87	0.64	5.49E-05
rs11243916	9	135675757	T	C	0.10	-0.71	0.18	1.20E-05
rs1887389	9	19848344	T	G	0.75	0.41	0.10	6.08E-05
rs4742301	9	7065665	A	G	0.80	0.53	0.12	2.48E-06
rs74446676	9	8870869	T	C	0.04	-1.17	0.35	6.06E-05
rs11016130	10	130013682	T	A	0.24	0.40	0.09	1.70E-05
rs10400076	10	18802698	A	T	0.36	-0.35	0.09	7.18E-05
rs112966083	10	6613974	T	C	0.02	1.02	0.23	4.65E-05

rs76843061	10	75538382	T	C	0.02	-4.03	1.74	7.46E-06
rs16935806	10	80074666	G	T	0.06	0.61	0.14	5.48E-05
rs111555687	10	91579667	C	T	0.02	0.91	0.21	4.90E-05
rs80216562	11	128322602	A	G	0.04	0.76	0.17	2.30E-05
rs186206154	11	5186285	T	C	0.04	0.79	0.19	8.10E-05
rs163693	11	72360787	C	T	0.83	-0.44	0.10	2.03E-05
rs61885383	11	79009802	A	G	0.15	0.47	0.10	9.95E-06
rs12272069	11	9419871	G	A	0.08	0.60	0.13	1.31E-05
rs115810645	12	114261795	C	T	0.02	-2.01	0.73	8.35E-05
rs12425066	12	124754623	A	C	0.10	0.51	0.12	5.09E-05
rs61317266	12	13632087	G	T	0.10	0.51	0.12	5.62E-05
rs141363582	12	2310896	T	C	0.02	-2.41	0.91	7.76E-05
rs12321522	12	27165684	A	G	0.07	-0.80	0.22	3.48E-05
rs73083471	12	32766299	T	G	0.10	-0.66	0.17	3.48E-05
rs9788221	12	45543717	T	C	0.15	-0.53	0.14	3.02E-05
rs17782033	12	67805364	G	A	0.05	-1.09	0.30	3.13E-05
rs73138189	12	68691953	A	C	0.16	-0.54	0.13	1.37E-05
rs79645696	12	78830953	A	G	0.05	-0.97	0.29	9.34E-05
rs7999854	13	111250160	T	C	0.11	-0.59	0.16	9.38E-05
rs79015068	13	46933101	C	T	0.05	0.73	0.17	7.61E-05
rs149354575	13	65525753	C	T	0.02	1.10	0.24	1.98E-05
rs7154465	14	92983117	G	T	0.62	-0.35	0.08	2.99E-05
rs16961323	15	48959082	A	G	0.06	-0.94	0.25	2.06E-05
rs7182840	15	86796682	G	T	0.54	0.33	0.08	6.83E-05
rs11637922	15	88200924	G	T	0.43	0.36	0.08	1.14E-05
rs569734	16	26808493	T	A	0.03	0.81	0.19	7.41E-05
rs11860162	16	57320684	T	C	0.41	0.35	0.09	7.48E-05
rs12324937	16	75014160	T	C	0.07	0.57	0.14	7.37E-05
rs72802206	16	83227970	A	G	0.18	0.40	0.10	8.63E-05
rs11870485	17	2705901	A	C	0.16	-0.53	0.14	7.20E-05
rs2735475	17	34258007	C	A	0.16	-0.60	0.14	2.18E-06
rs17686238	17	43417273	T	G	0.11	0.48	0.12	7.80E-05
rs874369	17	64890122	A	G	0.32	-0.38	0.09	4.31E-05
rs9911654	17	69660412	C	T	0.08	-0.70	0.20	9.91E-05
rs77772909	18	1423312	C	T	0.02	-2.22	0.76	2.13E-05
rs62100750	18	33434387	C	T	0.09	-0.69	0.19	6.99E-05
rs35766299	18	52752045	A	G	0.12	-0.57	0.16	9.65E-05
rs17819978	18	72440801	C	T	0.14	-0.58	0.15	2.17E-05
rs62105797	19	30145601	C	T	0.12	-0.60	0.16	3.22E-05
rs112769502	19	50130006	T	C	0.07	0.64	0.14	8.46E-06
rs111302614	19	5150243	A	C	0.09	-0.70	0.19	7.64E-05
rs6062957	20	61351085	A	G	0.48	0.38	0.08	2.86E-06
rs7267551	20	6932220	C	T	0.22	-0.43	0.11	6.93E-05

rs77993217	21	20965510	A	C	0.05	0.66	0.15	4.79E-05
rs112350442	21	43501421	A	G	0.06	-0.88	0.25	6.58E-05
rs79383759	22	48868007	A	T	0.09	0.60	0.13	1.87E-05

SNP: Single nucleotide polymorphism; CHR: Chromosome; BP: Base pair position; A1FREQ: Alle frequency of allele 1; Beta: Effect size of allele 1; SE: Standard error; P: P value

Supplementary Table 5. Detail on the missing data approach

To mitigate bias as a result of missing data, multiple imputation was used to estimate imputed prevalence and the crude and adjusted risk ratios presented in our paper (Rubin 1987, Rubin and Little 2002). We used a Fully Conditional Specification approach (van Buuren 2007) with -mi impute chained- in Stata version 16 (StataCorp, 2019).

Approach	Model and justification
A Fully Conditional Specification approach comprising 25 cycles of regression-switching which produced 100 imputed datasets with imputation sample stratified by sex.	Incomplete binary variables were imputed using logistic regression, with ordinal regression utilised for categorical variables. Depending on variable distributions we imputed continuous data either under a normal model or using prediction mean matching with ten nearest-neighbours. Auxiliary data were included in an attempt to improve the validity of the required Missing At Random (MAR) assumption (White, Royston et al. 2011). Auxiliary variables differed for each prediction equation and comprised strong correlates of the incomplete variable being imputed. For example, more-complete measurements collected at previous or later data-collection waves. Our chosen strategy was additionally based on the assumption that IQ was likely to be Missing Not At Random (MNAR) within the sample of cases with complete IQ. We therefore restricted our final sample to include those with complete IQ at age 8 and BID data due to a lack of good auxiliary data for these incomplete measure variables. The imputation sample was therefore restricted to 5,193 participants (2,734 females and 2,459 males).

Supplementary Table 6. Cross tabulations of body image at age 10 (mean age: 10.7 years) on body size categories derived from body mass index at age 7 (mean age: 7.6 years) (n = 5,193)

Body size (based on BMI measurements)	Body image dissatisfaction		
	Desire to be larger (row %)	Satisfied (row %)	Desire to be smaller (row %)
Under weight	126 (32.4)	253 (65.0)	10 (2.6)
Normal weight	287 (7.6)	2802 (74.3)	682 (18.1)
Overweight [a]	<5 (<1.0%)	~230 (~35.0)	~430 (~64.0)

[a] – Cell counts <5 cannot be presented to avoid revealing identifying information about participants. Exact values for the other columns are therefore presented as approximate values

Two combinations of responses are rare (specifically, those experiencing dissatisfaction (desire to be larger) whilst being larger and those experiencing dissatisfaction (desire to be smaller) whilst being smaller.

Supplementary Table 7. Distribution of body size at age 7 (mean age: 7.6 years) in those experiencing body image dissatisfaction at age 10 (mean age: 10.7 years) (imputed data, n = 5,193)

		Total (imputed data, n = 5,193)			Females (imputed data, n = 2,734)			Males (imputed data, n = 2,459)		
Variable	Category	Total, No. (not imputed)	No. (row %) of adolescence with outcome	Imputed prevalence (95% CI)	Total, No. (not imputed)	No. (row %) of adolescence with outcome	Imputed prevalence (95% CI)	Total, No. (not imputed)	No. (row %) of adolescence with outcome	Imputed prevalence (95% CI)
Outcome comparison: Body image dissatisfaction - desire to be smaller versus satisfied with body image										
Body size	Normal weight	3771	682 (18.1%)	18.2% (17.1%-19.5%)	1911	436 (22.8%)	22.9% (21.1%-24.8%)	1860	246 (13.2%)	13.5% (12.0%-15.1%)
	Underweight	389	10 (2.6%)	3.0% (1.7%-5.4%)	194	6 (3.1%)	3.9% (1.9%-8.0%)	195	<5 (~2.0%) [a]	2.2% (0.8%-5.7%)
	Overweight	669	432 (64.6%)	64.8% (61.4%-68.4%)	427	278 (65.1%)	65.3% (61.1%-70.0%)	242	154 (63.6%)	63.8% (58.2%-70.1%)
Outcome comparison: Body image dissatisfaction - desire to be larger versus satisfied with body image										
Body size	Normal weight	3771	287 (7.6%)	7.6% (6.8%-8.5%)	1911	106 (5.6%)	5.5% (4.6%-6.5%)	1860	181 (9.7%)	9.8% (8.6%-11.2%)
	Underweight	389	126 (32.4%)	32.4% (28.1%-37.3%)	194	58 (29.9%)	29.2% (23.6%-36.1%)	195	68 (34.9%)	35.6% (29.6%-42.9)
	Overweight	669	<5 (<1.0%) [a]	0.6% (0.2%-1.5%)	427	<5 (<1.0%) [a]	0.4% (0.1%-1.8%)	242	<5 (<1.0%) [a]	0.8% (0.2%-3.1%)

[a] – Cell counts <5 cannot be presented to avoid revealing identifying information about participants. Exact values for the other columns are therefore presented as approximate values

Supplementary Table 8A. Bidirectional univariable Mendelian randomization analyses for childhood body size at age 10 (mean age: 10 years) onto body image dissatisfaction (desire to be smaller) (at age 10 (mean age: 10.7 years) and body image dissatisfaction (desire to be smaller) on childhood body size

Exposure	Outcome	nSNPs*	Risk difference (95% CI)	Standard Error	*P* value	Method
Childhood body size (UK Biobank)	BID (desire to be smaller) (ALSPAC)	240	2.785 (2.147-3.423)	0.326	1.194×10^{-17}	MR-RAPS
Childhood body size (UK Biobank)	BID (desire to be smaller) (ALSPAC)	240	2.767 (2.154-3.381)	0.313	9.575×10^{-19}	IVW
Childhood body size (UK Biobank)	BID (desire to be smaller) (ALSPAC)	240	3.086 (2.076-4.096)	0.515	2.095×10^{-9}	Weighted median
Childhood body size (UK Biobank)	BID (desire to be smaller) (ALSPAC)	240	3.262 (1.952-4.572)	0.668	1.934×10^{-6}	MR Egger
BID (desire to be smaller) (ALSPAC)	Childhood body size (UK Biobank)	138	0.002 (-0.001-0.002)	0.001	0.817	MR-RAPS
BID (desire to be smaller) (ALSPAC)	Childhood body size (UK Biobank)	138	0.002 (-0.001-0.005)	0.002	0.147	IVW
BID (desire to be smaller) (ALSPAC)	Childhood body size (UK Biobank)	138	0.000 (-0.001-0.002)	0.001	0.827	Weighted median
BID (desire to be smaller) (ALSPAC)	Childhood body size (UK Biobank)	138	-0.006 (-0.013-0.001)	0.004	0.118	MR Egger

* nSNPs = number of single nucleotide polymorphisms

Supplementary Table 8B. Bidirectional univariable Mendelian randomization analyses for childhood body size at age 10 (mean age: 10 years) onto body image dissatisfaction (desire to be larger) (at age 10 (mean age: 10.7 years) and body image dissatisfaction (desire to be larger) on childhood body size

Exposure	Outcome	nSNPs*	Risk difference (95% CI)	Standard Error	*P* value	Method
Childhood body size (UK Biobank)	BID (desire to be larger) (ALSPAC)	240	-2.931 (-3.885- -1.977)	0.487	1.729×10^{-9}	MR-RAPS
Childhood body size (UK Biobank)	BID (desire to be larger) (ALSPAC)	240	-2.943 (-3.857- -2.028)	0.467	2.850×10^{-10}	IVW
Childhood body size (UK Biobank)	BID (desire to be larger) (ALSPAC)	240	-3.394 (-5.025- -1.764)	0.832	4.508×10^{-5}	Weighted median
Childhood body size (UK Biobank)	BID (desire to be larger) (ALSPAC)	240	-3.978 (-5.918- -2.037)	0.990	7.886×10^{-5}	MR Egger
BID (desire to be larger) (ALSPAC)	Childhood body size (UK Biobank)	130	0.000 (-0.001-0.001)	0.001	0.582	MR-RAPS
BID (desire to be larger) (ALSPAC)	Childhood body size (UK Biobank)	130	-0.001 (-0.002-0.001)	0.001	0.273	IVW
BID (desire to be larger) (ALSPAC)	Childhood body size (UK Biobank)	130	0.000 (-0.001-0.001)	0.001	0.506	Weighted median
BID (desire to be larger) (ALSPAC)	Childhood body size (UK Biobank)	130	0.001 (-0.001-0.003)	0.001	0.586	MR Egger

* nSNPs = number of single nucleotide polymorphisms

Supplementary Table 9. Prospective association between body size at age 7 (mean age: 7.6 years) and body image at age 10 (mean age: 10.7 years) (imputed data, n = 5,193)

		Females (imputed data, n = 2,734)		Males (imputed data, n = 2,459)	
Variable	Category	Crude multinomial odds ratio (95% CI)	Adjusted multinomial odds ratio (95% CI)*	Crude multinomial odds ratio (95% CI)	Adjusted multinomial odds ratio (95% CI)*
Outcome comparison: Body image dissatisfaction - desire to be smaller versus satisfied with body image					
Body size	Normal weight	1 [Reference]	1 [Reference]	1 [Reference]	1 [Reference]
	Underweight	0.18 (0.0.9, 0.39)	0.19 (0.09, 0.41	0.20 (0.07, 0.54)	0.19 (0.07, 0.53)
	Overweight	6.00 (4.79, 7.50)	5.45 (4.32, 6.88)	10.28 (7.68, 13.76)	9.62 (7.09, 13.05)
Outcome comparison: Body image dissatisfaction - desire to be larger versus satisfied with body image					
Body size	Normal weight	1 [Reference]	1 [Reference]	1 [Reference]	1 [Reference]
	Underweight	5.73 (3.99, 8.24)	5.77 (3.91, 8.50)	4.47 (3.22, 6.20)	4.50 (3.19, 6.35)
	Overweight	0.17 (0.04, 0.71)	0.14 (0.03, 0.60)	0.17 (0.04, 0.71)	1. 0.17 (0.04, 0.72)
	P value	<0.0001	<0.0001	<0.0001	<0.0001

*Adjusted for birthweight, IQ, maternal education, ethnicity, father absence, weekly income, parent social class, maternal age, maternal BMI, the Edinburgh Postnatal Depression Scale (32 weeks gestation & 21 months), and stressful life events during childhood assessed using a questionnaire completed by mothers recording whether the child had experienced any of 16 upsetting events since the child was 5 years

Supplementary Figure 1. Stunkard figure rating scales questionnaire administered to children (mean age: 10.7 years)

CCH File – Section B

PAGE 4 OF THE FEMALE QUESTIONNAIRE

Section B: Pictures of different girls

B1. Here are pictures of 5 girls. Please put a tick in the box under the drawing that is most like you:

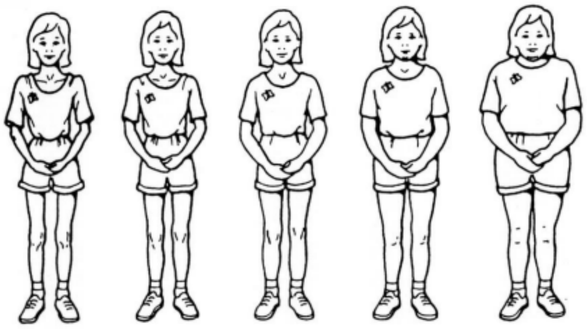

a) ☐1 ☐2 ☐3 ☐4 ☐5

b) Now tick the box that you would most like to be. This can be the same one as in your answer above.

☐1 ☐2 ☐3 ☐4 ☐5

CCH File – Section B

PAGE 4 OF THE MALE QUESTIONNAIRE

Section B: Pictures of different boys

B1. Here are pictures of 5 boys. Please put a tick in the box under the drawing that is most like you:

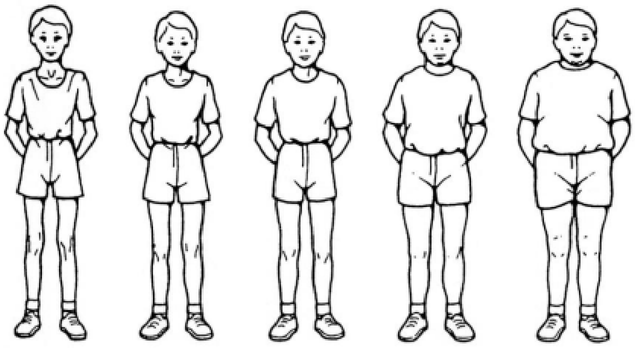

a) ☐1 ☐2 ☐3 ☐4 ☐5

b) Now tick the box that you would most like to be. This can be the same one as in your answer above.

☐1 ☐2 ☐3 ☐4 ☐5

Supplementary Figure 2. Flowchart delineating the three steps involved in conducting analyses for this research

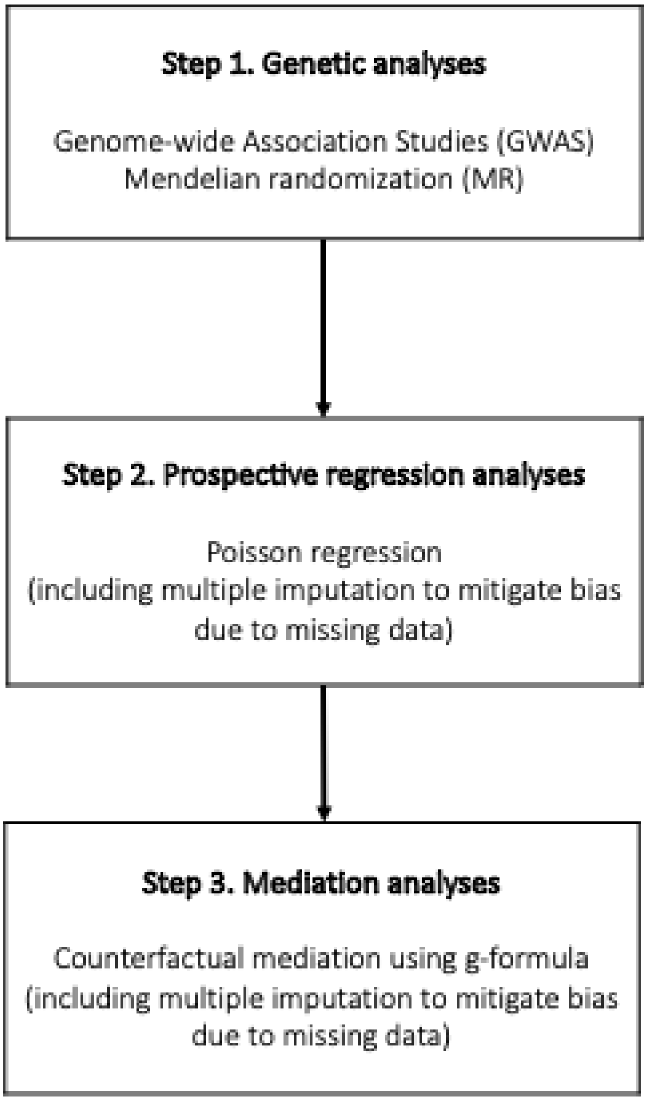

Supplementary Figure 3A. Manhattan plot displaying the results of the genome-wide association study (GWAS) for body image dissatisfaction (desire to be smaller)

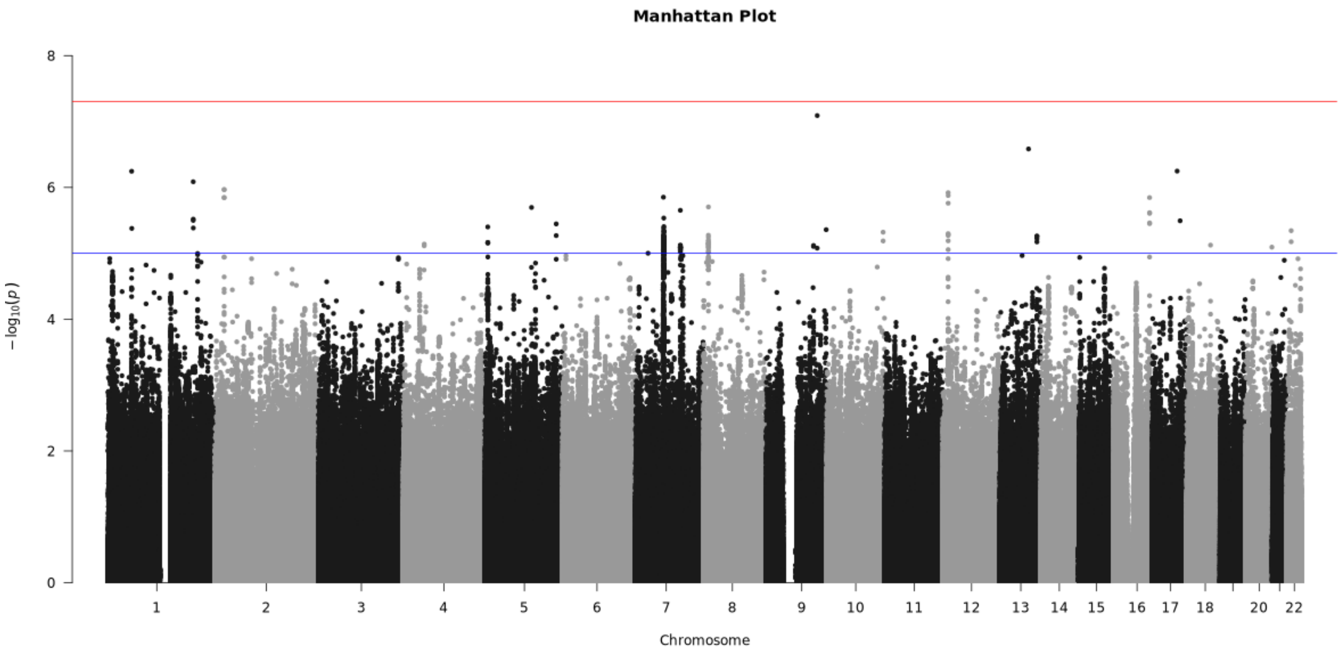

Horizontal lines are drawn at - log10(1 × 10^{-5}) for "suggestive associations" in blue and - log10(5 × 10^{-8}) for the "genome-wide significant" threshold in red.

Supplementary Figure 3B. QQ plot illustrating the observed vs. expected -log10(p-values) from the GWAS for body image dissatisfaction (desire to be smaller)

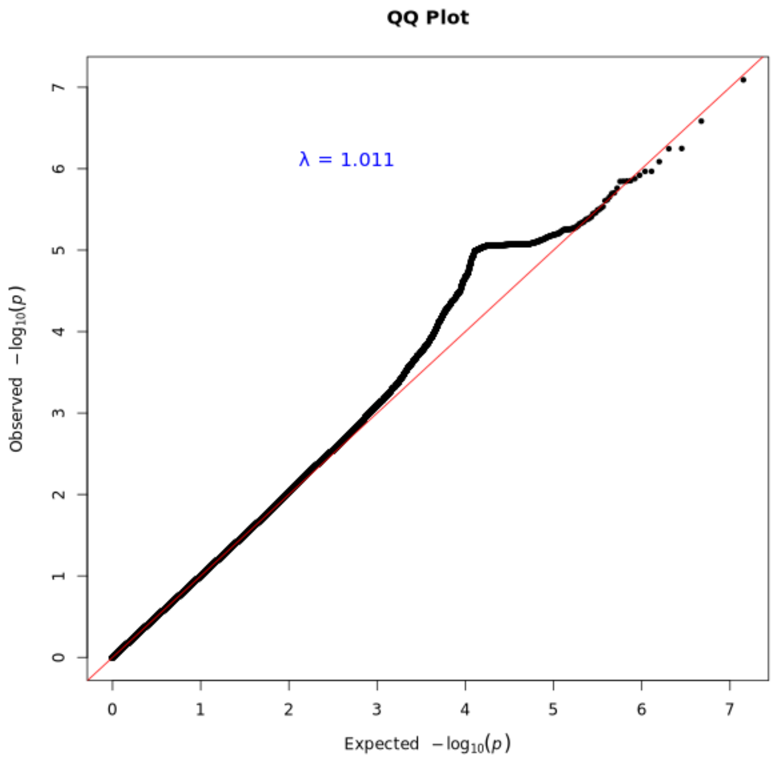

The red line represents the null hypothesis of no association. The lambda (λ) value, indicating the genomic inflation factor, is calculated to be 1.011, suggesting minimal population stratification or other confounding factors.

Supplementary Figure 4A. Manhattan plot displaying the results of the genome-wide association study (GWAS) for body image dissatisfaction (desire to be larger)

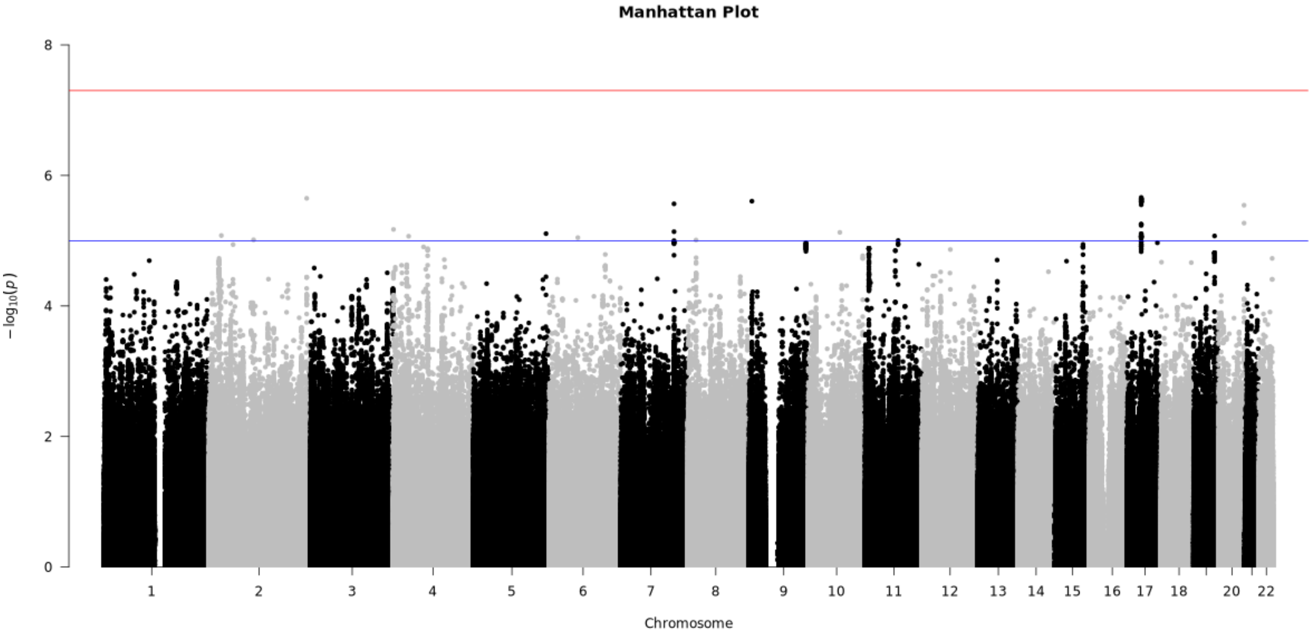

The x-axis represents the chromosomes, while the y-axis represents the -log10(p-values) of the SNPs. Horizontal lines are drawn at -log10(1×10^{-5}) for "suggestive associations" in blue and -log10(5×10^{-8}) for the "genome-wide significant" threshold in red.

Supplementary Figure 4B. QQ plot illustrating the observed vs. expected -log10(p-values) from the GWAS for body image dissatisfaction (desire to be larger)

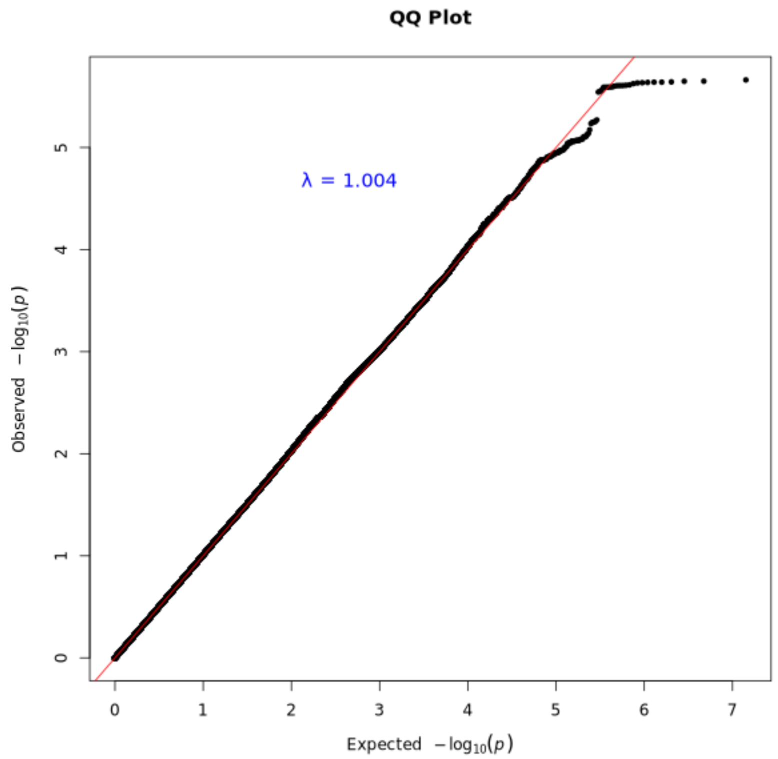

The red line represents the null hypothesis of no association. The lambda (λ) value, indicating the genomic inflation factor, is calculated to be 1.004, suggesting minimal population stratification or other confounding factors.

References

Bowes, L., C. Joinson, D. Wolke and G. Lewis (2015). "Peer victimisation during adolescence and its impact on depression in early adulthood: prospective cohort study in the United Kingdom." BMJ : British Medical Journal **350**: h2469.

Cole, T. J., M. C. Bellizzi, K. M. Flegal and W. H. Dietz (2000). "Establishing a standard definition for child overweight and obesity worldwide: international survey." Bmj **320**(7244): 1240-1243.

Cole, T. J., K. M. Flegal, D. Nicholls and A. A. Jackson (2007). "Body mass index cut offs to define thinness in children and adolescents: international survey." Bmj **335**(7612): 194.

Cox, J. L., J. M. Holden and R. Sagovsky (1987). "Detection of postnatal depression. Development of the 10-item Edinburgh Postnatal Depression Scale." Br J Psychiatry **150**: 782-786.

Fisher, H. L., A. Schreier, S. Zammit, B. Maughan, M. R. Munafò, G. Lewis and D. Wolke (2012). "Pathways Between Childhood Victimization and Psychosis-like Symptoms in the ALSPAC Birth Cohort." Schizophrenia Bulletin **39**(5): 1045-1055.

Harter, S. (1985). "Self-perception profile for children." Hispanic Journal of Behavioral Sciences.

Kann, L., C. W. Warren, W. A. Harris, J. L. Collins, B. I. Williams, J. G. Ross and L. J. Kolbe (1996). "Youth Risk Behavior Surveillance—United States, 1995." Journal of School Health **66**(10): 365-377.

Madge, N., A. Hewitt, K. Hawton, E. J. de Wilde, P. Corcoran, S. Fekete, K. van Heeringen, D. De Leo and M. Ystgaard (2008). "Deliberate self-harm within an international community sample of young people: comparative findings from the Child & Adolescent Self-harm in Europe (CASE) Study." J Child Psychol Psychiatry **49**(6): 667-677.

Rubin, D. B. (1987). Multiple imputation for nonresponse in surveys. New York, Wiley.

Rubin, D. B. and R. J. Little (2002). Statistical analysis with missing data. Hoboken, NJ, J Wiley & Sons.

van Buuren, S. (2007). "Multiple imputation of discrete and continuous data by fully conditional specification." Stat Methods Med Res **16**(3): 219-242.

Warne, N., J. Heron, B. Mars, P. Moran, A. Stewart, M. Munafò, L. Biddle, A. Skinner, D. Gunnell and H. Bould (2021). "Comorbidity of self-harm and disordered eating in young people: Evidence from a UK population-based cohort." Journal of Affective Disorders **282**: 386-390.

Warne, N., J. Heron, A. von Gontard and C. Joinson (2023). "Mental health problems, stressful life events and new-onset urinary incontinence in primary school-age children: a prospective cohort study." European Child & Adolescent Psychiatry.

Wechsler, D. (1991). "Wechsler Intelligence Scale for Children Third Edition manual."

White, I. R., P. Royston and A. M. Wood (2011). "Multiple imputation using chained equations: Issues and guidance for practice." Stat Med **30**(4): 377-399.

Wolke, D., S. Woods, K. Stanford and H. Schulz (2001). "Bullying and victimization of primary school children in England and Germany: prevalence and school factors." Br J Psychol **92**(Pt 4): 673-696.
